# Integrated computational prediction and experimental validation identifies promiscuous T cell epitopes in the proteome of *Mycobacterium bovis*

**DOI:** 10.1099/mgen.0.000071

**Published:** 2016-08-25

**Authors:** Damien Farrell, Gareth Jones, Christopher Pirson, Kerri Malone, Kevin Rue-Albrecht, Anthony J. Chubb, Martin Vordermeier, Stephen V. Gordon

**Affiliations:** ^1^​ School of Veterinary Medicine, University College Dublin, Dublin D4, Ireland; ^2^​ Department of Bacteriology, Animal and Plant Health Agency, New Haw, Surrey KT15 3NB, UK; ^3^​ School of Agriculture and Food Science, University College Dublin, Dublin 4, Ireland; ^4^​ School of Medicine, University College Dublin, Dublin D4, Ireland; ^5^​ Conway Institute of Biomolecular and Biomedical Science, University College Dublin, Dublin D4, Ireland; ^6^​ School of Biomolecular and Biomedical Science, University College Dublin, Dublin 4, Ireland

**Keywords:** *Mycobacterium bovis*, Tuberculosis, epitope, MHC, bovine

## Abstract

The discovery of novel antigens is an essential requirement in devising new diagnostics or vaccines for use in control programmes against human tuberculosis (TB) and bovine tuberculosis (bTB). Identification of potential epitopes recognised by CD4^+^ T cells requires prediction of peptide binding to MHC class-II, an obligatory prerequisite for T cell recognition. To comprehensively prioritise potential MHC-II-binding epitopes from *Mycobacterium bovis*, the agent of bTB and zoonotic TB in humans, we integrated three binding prediction methods with the *M. bovis*proteome using a subset of human HLA alleles to approximate the binding of epitope-containing peptides to the bovine MHC class II molecule BoLA-DRB3. Two parallel strategies were then applied to filter the resulting set of binders: identification of the top-scoring binders or clusters of binders. Our approach was tested experimentally by assessing the capacity of predicted promiscuous peptides to drive interferon-γ secretion from T cells of *M. bovis* infected cattle. Thus, 376 20-mer peptides, were synthesised (270 predicted epitopes, 94 random peptides with low predictive scores and 12 positive controls of known epitopes). The results of this validation demonstrated significant enrichment (>24 %) of promiscuously recognised peptides predicted in our selection strategies, compared with randomly selected peptides with low prediction scores. Our strategy offers a general approach to the identification of promiscuous epitopes tailored to target populations where there is limited knowledge of MHC allelic diversity.

## Data Summary

1. All computational work described here was implemented using custom Python scripts. The majority of code is implemented as a Python library called *epitopepredict* and is available at https://github.com/dmnfarrell/epitopepredict under the Apache license.
2. Extensive use was made of the IPython (Jupyter) notebook environment to generate publication plots and allow the workflow to be reproduced. The notebook and intermediate data files for generating the plots are available at https://github.com/dmnfarrell/gordon-group/tree/master/antigen-mining. This includes the lists of binders generated from each prediction method for the entire proteome.
3. Individual prediction results for any protein in the *M. bovis* genome can be visualised at http://enzyme.ucd.ie/epitopemap using the Epitopemap web application (Farrell & Gordon, 2015). Login details are given in Appendix S1.

## Impact Statement

The discovery of new antigens is a prerequisite in devising new diagnostics and vaccines for use in infectious disease control programmes. Protein antigens are digested by the cellular machinery into smaller fragments called peptides with a small fraction of these peptides, called epitopes, then presented to T cells on the surface of cells (designated antigen-presenting cells) in a complex composed of T cell receptor (TCR), and the peptide bound to a major histocompatibility complex (MHC) protein molecule. Computer algorithms can scan an entire bacterial genome and predict which parts of the protein sequences are potential MHC-binders. Such fast-track screening is needed because it is impossible to experimentally test all the possible peptides in a pathogen genome (e.g. for *Mycobacterium bovis* >1 million peptides). Using a computational strategy we selected 376 peptides to be synthesized and tested in *M. bovis*-infected animals. Our computational pipeline successfully enriched for peptides containing promiscuous epitopes, far in excess of what would be expected by chance. Our work increases considerably the hitherto known set of potential *M. bovis* antigens, and proves the utility of computational approaches to T-cell antigen-identification for infectious diseases.

## Introduction

*Mycobacterium bovis* is the main causative pathogen of bovine tuberculosis (bTB), which remains a significant economic and animal welfare concern to global agriculture, as well as representing a zoonotic risk to human health. Control of bTB currently relies on the identification of infected cattle using a crude mix of bacterial proteins (termed purified protein derivative, or PPD) that are used in a skin test; animals that show evidence of *M. bovis* infection are deemed ‘reactors’ and culled. The use of such crude antigen preparations presents problems in terms of standardisation, potency testing and specificity. Vaccination is not currently used as a control strategy as the only available vaccine, BCG, compromises the skin test by confounding the identification of vaccinated vs infected animals (Hogarth *et al.*, 2006). A new generation of bTB disease control tools is needed that exploits advances in ‘omics technologies and allied computational approaches to transcend the limitations of current diagnostics.

The predominant protective and immunopathological responses against *M. bovis* rely on cellular immune responses involving mainly CD4^+^ T cells; thus the rational development of new immunodiagnostics and vaccines relies on knowledge of the underlying T cell responses. For example, diagnostic antigen prototypes that will work in the face of BCG vaccination have been based on proteins whose genes are deleted from BCG (e.g. ESAT-6 or CFP-10) or are not secreted by BCG (e.g. Rv3615c) (Sidders *et al.*, 2008). A parallel approach is to improve BCG vaccine efficacy by boosting or supplementing its efficacy with subunit vaccines such as virally-vectored vaccines that encode T-cell antigens (Vordermeier *et al.*, 2011; Waters *et al.*, 2012). Both approaches rely on the identification of distinct antigen repertoires. Identification of antigens or epitopes currently relies on direct testing of purified proteins or peptides in interferon-gamma release assays (IGRAs), or other tests that probe T-cell recognition, using populations of immune cells from infected animals. Given the 3961 annotated protein-encoding genes in the *M. bovis* genome, and the millions of potential epitopes, such an empirical approach to comprehensive, genome-wide antigen discovery is prohibitively expensive (Lindestam Arlehamn & Sette, 2014). Therefore, antigen discovery for the purposes of vaccine or diagnostic test development has in the past been done largely using knowledge-based rationale, i.e. according to known functional classification; this has a tendency to be self-reinforcing (Geluk *et al.*, 2014) and concentrates on specific subsets of the proteome. A pre-screen where potential T-cell epitopes could be identified based on their predicted binding to the MHC complex would be an ideal filter to identify peptides that could then be synthesised and screened in infected animals. Accurate prediction of the peptides that will form antigenic epitopes is therefore essential for genome-wide antigen discovery.

Epitope prediction methods have thus far concentrated largely on the MHC–peptide binding step since this is most amenable to computational analysis (Nielsen *et al.*, 2010; Wang *et al.*, 2008). These methods predict the binding affinity of a peptide sequence to a specific MHC molecule. A variety of data-driven approaches have been used (Patronov & Doytchinova, 2013). All such methods vary in accuracy over MHC loci and alleles, largely depending on the availability of binding data. A given peptide may also be able to bind to multiple alleles, often described as 'binding promiscuity'. In the context of eliciting an immune response, high binding affinity and promiscuity implies responses in a large proportion of populations with diverse MHC genotypes. There may also only be a few dominant peptides in a given protein responsible for its antigenicity in much of the population. The challenge in designing both epitope-based vaccines and diagnostics is to find an optimal epitope repertoire that provides broad population coverage. Results from binding-prediction algorithms must be treated carefully if trying to extract immunogenic epitopes from many possible peptides (Chaves *et al.*, 2012). Cut-offs can be raised at the cost of missing potential valuable antigenic peptides; on the other hand, too low a cut-off yields a larger number of synthetic peptides to test, many of which will be negative. There is therefore a trade-off between discovery and costs of experimental validation. Some current prediction approaches are detailed and compared in a review by Schubert *et al.* (2013a).

Specific strategies for filtering candidate T cell epitopes from the large number of possible peptides will vary depending on the desired outcome (Lundegaard *et al.*, 2010). The simplest method is to rank peptides by binding affinity and select the top scorers; this is however problematic due to predictor accuracy. Several recent studies (Santos *et al.*, 2013; Zhang *et al.*, 2008; Zvi *et al.*, 2012) have instead used the concept of epitope density to find likely regions of antigenicity within protein sequences. These computational screens calculated clusters or hotspots of epitopes in regions along the sequence and ranked them by a density-related metric. Furthermore Gaseitsiwe *et al.* (2010) calculated the number of experimentally derived epitopes per total peptides in each protein as a criterion to rank 61 *M. tuberculosis* proteins for potential antigenicity. There is empirical evidence that these cluster-based approaches are valid: MHC class II-binding T cell epitopes have been observed to occur in clusters of up to 25 amino acids in length (De Groot & Martin, 2009). Thus MHC class II T cell epitope clusters may represent regions of the protein with high affinity across multiple MHC alleles, ideal for promiscuous epitope prediction.

Prediction tools have been used commonly to study small, manageable subsets of proteins selected on the basis of known properties, and have largely focussed on human MHC alleles [human leukocyte antigens (HLA's)] (Gurung *et al.*, 2012; Zvi *et al.*, 2011). Computational methods for T-cell epitope identification have been applied to the *Mycobacterium tuberculosis* genome (Lindestam Arlehamn *et al.*, 2013; Santos *et al.*, 2013; Zvi *et al.*, 2008) with some success. These latter studies use a mixture of knowledge-based and unbiased methods; however, truly unbiased methods have the most potential for novel antigen discovery.

The MHC loci have a similar structure among mammalian species, and in cattle are called the bovine leukocyte antigen (BoLA) genes. The BoLA class II region consists of one DRA, at least three DRB loci and multiple DQA and DQB genes. The extensive diversity of MHC genes in domestic cattle demonstrates the dynamic nature of the MHC region despite controlled breeding and population bottlenecks (Mikko & Andersson, 1995). BoLA-DRB3.2, which is the second DRB3 exon, is responsible for the β1 domain of the only widely expressed DRB gene in cattle (Behl *et al.*, 2012). Numerous studies in diverse cattle breeds have reported a high degree of polymorphism at this locus and the Immuno Polymorphism Database (IPD-MHC) (Robinson *et al.*, 2010) for cattle currently contains 130 alleles for this gene. The DQA and DQB loci are also polymorphic.

Our objective was to predict a set of *M. bovis* epitopes that reflect the allele-specific immune response to this pathogen. Since experimental binding data specific to BoLA-DRB3 alleles is not available, we used pan-computational methods to approximate predictions based on already known HLA-DR alleles. 'Pan' approaches are designed to allow methods trained on known alleles (those with available binding data sets) to be extrapolated to unknown alleles. Despite very limited validation, it has also been shown that MHC class II pan-specific predictions can be applied to cattle (Jones *et al.*, 2011; Vordermeier *et al.*, 2003) using human alleles. Our approach provided a significant enrichment for identification of T-cell epitopes, and underlines the potential of computational methods to accelerate antigen identification.

## Methods

### MHC class II binding-prediction tools

For large-scale screening of epitopes in an entire proteome binding-prediction methods must be rapid since a calculation must be made for each potential sequence. In *M bovis* the total number of 20-mer peptides is 1 228 066 in 3961 proteins. Even allocating two weeks to run the entire genome requires that each prediction takes at most 1.8 s per peptide per cpu; this then has to be repeated for each allele.

Two MHC class II binding-prediction methods that fit the speed and accuracy requirements were used in this study:

1. TEPITOPEpan (Zhang *et al.*, 2012) is a position-specific scoring matrix (PSSM)-based algorithm (Raddrizzani & Hammer, 2000). It uses 11 scoring matrices derived from combinatorial competitive binding assays on 11 HLA-DR alleles (Sturniolo *et al.*, 1999). This method covers 700 HLA-DR molecules with unknown binding specificities based on pocket similarity (Zhang *et al.*, 2009) to the original set of 11 library sequences. We have independently implemented this algorithm in Python and it is available as part of the epitope*predict* library.
2. NetMHCIIpan (Nielsen *et al.*, 2008) is an artificial neural network algorithm trained on binding data for multiple MHC-II alleles. Predictions are now extended to all HLA-DR, DQ and DP known sequences as from version 3.0 (Karosiene *et al.*, 2013).

For MHC class I binding prediction the IEDB prediction tools were used with the ‘recommended’ option (Kim *et al.*, 2012).

### Cut-offs for predictors

A typical approach to binder selection is to select the top nth percentile per protein rather than using an absolute threshold value; however for whole-proteome studies this is likely to introduce multiple false positives from peptides in proteins that would otherwise score very low globally. We therefore adopted a method of global standardization of the score over the entire proteome, similar to that used by Bremel & Homan (2010) and others, by setting a global cut-off based on the top 3 % of scores from the entire proteome. In addition, some alleles have a significantly higher score distribution and will dominate the results if a uniform score cut-off is applied; this applies in general to MHC-binding predictors. Thus we applied a separate global cut-off per allele so that low-scoring alleles would be better represented when calculating our promiscuity criterion. This approach is consistent with recent work by. Paul *et al.* (2013) regarding allele-specific thresholds in MHC-I prediction.

### Comparison of alleles

**Fig. 1.**
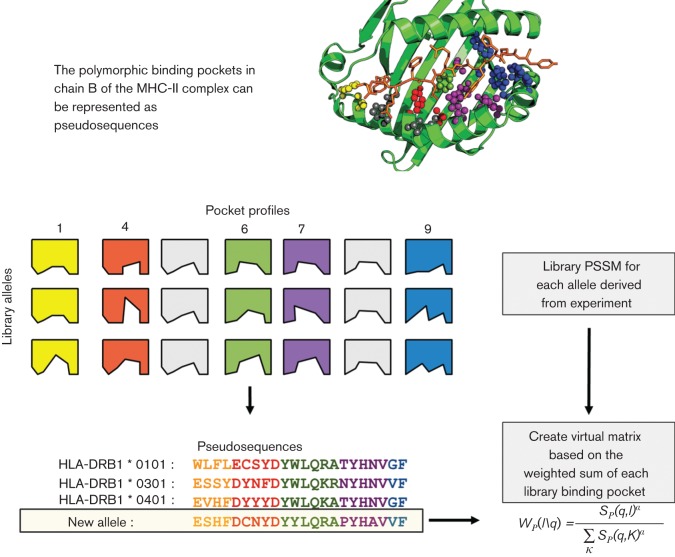
Pocket profile concept for MHC binding prediction. The polymorphic binding pockets in chain B of the MHC-II complex. This concept is used in TEPITOPEpan to treat alleles being composed of independent, modular pockets that can be extrapolated to other alleles with no binding data. The weight between query pocket *W* with query pseudo-sequence *q*, and a pocket in the library, with pseudo-sequence *l* is calculated as shown. *S_p_(q,l)* is the sequence similarity score. *K* denotes a sum over the entire pocket library. *α* is a positive parameter that determines the range of similarity scores that give high weights.

The available list of all BoLA-DRB3 alleles was downloaded from the IPD-MHC database (https://www.ebi.ac.uk/ipd/mhc/) and compared with known HLA-DR alleles from the IMGT/HLA database (https://www.ebi.ac.uk/ipd/imgt/hla/) (Robinson *et al.*, 2013). A multiple-sequence alignment was used to generate a set of pseudo-sequences, representing the allele using only the polymorphic residues in the binding groove of the MHC molecule. In order to determine the valid overlap between human and bovine alleles, the pseudo-sequence distance to each Tepitope library allele was calculated using the method of Nielsen *et al.* (2008). The concept of pocket profiles and pseudo-sequence is illustrated in Fig. 1.

### Epitope selection approaches

**Fig. 2.**
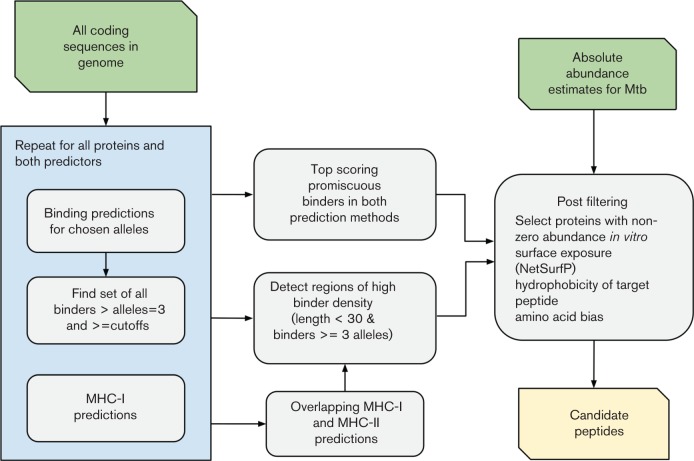
Schemes for peptide selection used in this study. In all selection strategies only binders above the cut-off in at least three alleles were considered. We chose 92 peptides from the top-scoring binders strategy and 176 from the binder clusters strategy for validation.

We used two contrasting strategies to select peptide candidates from the large list of potential binders that is summarised in Fig. 2. We applied both MHC-II binding-prediction methods over the whole proteome for eight HLA alleles (detailed in results). In all cases a binding promiscuity criterion was applied to binders whereby only predicted binders present across multiple alleles (three or more) were considered. These lists of binders could then be used in each of the three selection strategies described below.

#### Top-Scoring Binders.

This strategy is a variation on the standard approach (Hammond & Klein, 2005) of using top-ranking binders, but applied across the proteome using allele-specific cut-offs. It is designed to capture a set of proteins enriched with epitopes by finding proteins containing multiple high-scoring binders. A binder must satisfy the promiscuity criterion in both prediction methods to be considered. For each protein the number of binders per unit length is calculated (binders per unit length is used to prevent large proteins with many binders dominating). Proteins are then ranked by the sum of binder score and binder/length ranking. The final 92 peptides chosen are the 20-mers covering the top shared binders in the most highly ranked proteins.

#### Binder Clusters.

This strategy is designed to detect broad areas of both promiscuity and high binder density in each sequence. The assumption underlying this method is that approximatley 20-mer peptides covering these regions will be more likely to yield at least one true positive epitope and hence elicit a T-cell response. As above, only high promiscuity binders are considered. For each protein the set of all binders above appropriate cut-offs and present in at least three alleles were passed to the DBSCAN algorithm, (Ester *et al.*, 1996), a density-based clustering method, to detect areas of high binder density. Though normally employed on higher-dimensional data DBSCAN was found to be an appropriate solution. Only a distance measure between members and a minimum number of members are required as parameters, values that were estimated empirically to exclude sparse areas of binders. We used a distance of ten and minimum of three binders. The results are a set of clusters for both prediction methods, ranked by number of binders per unit length. This has also been referred to as the ‘epitope density’ method (Santos *et al.*, 2013). It was applied using both netMHCIIpan and TEPITOPEpan and finding overlaps between these methods.

#### Random binders (control).

To generate a control set of peptides to compare with our filtering methods we chose a random selection of 20 mers from the total list of 74 525 high promiscuity binders. Half of these were taken from TEPITOPEpan and half from netMHCIIpan predictions. They did not need to be present in both prediction methods. The only other filter placed on this selection was that they be contained in proteins with non-zero abundance as measured in *M. tuberculosis* H37Rv cultures (described below).

We therefore had two distinct strategies along with a set of randomly selected controls, summarised in Table 1. We could then compare the results from each strategy by their success in discovering immunogenic epitopes.

**Table 1.** Summary of the two epitope selection strategies and control used in this study

Strategy	Summary
Top-scoring binders	Highest scoring MHC-II binders in at least three alleles with at least one overlapping binder in both prediction algorithms (netMHCIIpan and TEPITOPEpan).
Binder clusters	Detects regions of densest binders using both TEPITOPEpan and netMHCIIpan predictors. Uses only overlapping binders from both methods and at least one MHC-I binder. Results vary depending on the order in which predictors are selected.
Random binders (control)	Baseline method using random binders from the entire set of predicted binders. Half were taken from TEPITOPEpan and half from netMHCIIpan without requiring that there is any overlap between predictors.

All binders are defined as being present in at least three of the eight chosen HLA MHC-II alleles.

### Filtering with overlapping predictions

Due to the large *n*-mer sequence space over the whole proteome, all three strategies produce a very large number of potential epitopes. To enhance our chances of identifying true epitopes, a key part of our method is to select out epitope regions that overlap with the other prediction methods, both MHC-I and MHC-II. That is, we selected only top-scoring binders or binder clusters that have at least one high-promiscuity binder according to the other methods also. This narrowed down our lists considerably.

For both strategies we only selected those binders that overlapped with at least one high-promiscuity MHC-II 9-mer binder from the other prediction algorithm., i.e. if using netMHCIIpan we require at least one overlapping binder from TEPITOPEpan and vice versa. At least one MHC-I 9-mer and MHC-I 11-mer binder was also required for a binder cluster to be considered. These MHC-I binder overlaps were not required for the top-scoring binders method since these are relatively rare and would remove too many of the top-scoring candidates.

Other filters:

For both strategies candidate peptides were further filtered by using the following criteria:

- Verification with abundance data from proteomics. We utilised recent data on absolute abundance levels of proteins in an unfractionated mixed lysate of *M. tuberculosis* H37Rv cultures identified by Schubert *et al.* (2013b). Screening antigen candidates based on expression is not novel (Sidders *et al.*, 2008) but these concentrations represent, to our knowledge, the most comprehensive proteome-wide estimates of *M. tuberculosis* protein expression thus far. All proteins undetected using selected reaction monitoring (concentration = 0 and <2 peptides detected) in the Schubert *et al.* study were filtered out. Protein selection was not ranked or biased with respect to abundance.
- Length based filter – remove all sequences with host proteins greater than 400 amino acids in length. The rationale for this is to select for epitopes in antigens that would be easier to clone and express recombinant protein from, if required.
- Burial/exposure based on Netsurfp predict (Petersen *et al.*, 2009). Jørgensen *et al.* (2010) concluded that MHC class II ligands are significantly more exposed than other peptides in the same protein with similar predicted binding affinity. They used sequence-based prediction of solvent exposure to establish a bias that exposed peptide fragments are more likely to be presented by MHC-II molecules compared with affinity-matched peptides in the same protein. We used this information as a screen to exclude and peptides predicted to have more than 50 % buried residues.
- Existence of already characterised peptides. Evidence that a candidate peptide has already been characterised was checked using data in the IEDB (Sette *et al.*, 2005) and other studies. However none were found.
- Hydrophobicity of peptide. Since strongly hydrophobic sequences can affect the solubility of the peptide we removed all peptides with >60 % hydrophobic residues. This is consistent with the data for T cell epitopes in the IEDB.
- Amino acid bias. We found a strong bias in the netMHCIIpan clusters for R, V and L amino acids. The distribution is skewed in relation to that seen for known epitopes in the IEDB. All peptides with highly biased R, V and L sequence content were also removed.

### Peptide synthesis

Peptides were prepared in standard PepSet (MimoTopes, 2014) libraries by Mimotopes (Victoria, Australia) in three randomized batches of 94 each. These were received in dried-down form and solubilised using 20 µl of DMSO, and then 180 µl of RPMI to obtain a concentration of 5 mg ml^−1^. Individual peptides were used in T cell assays at a final concentration of 20 µg ml^−1^.

### Experimental animals

Heparinised blood samples or peripheral blood mononuclear cells (PBMC) were obtained from naturally infected, single intradermal cervical comparative tuberculin-test-positive TB-reactors from herds known to have bovine tuberculosis. All animals were housed at the Animal and Plant Health Agency, UK, at the time of blood sampling, and the procedures were conducted within the limits of a United Kingdom Home Office Licence under the Animal (Scientific Procedures) Act 1986, which were approved by the local ethical review committee.

### T-cell assays

Whole-blood aliquots (250 µl) from four TB-reactor animals were added in duplicate to the peptides in 96-well plates, which were then incubated at 37 °C in the presence of 5 % CO_2_ for 24 h, after which plasma supernatants were harvested and stored at −80 °C until required. Cryopreserved PBMC from seven TB-reactor animals were thawed, re-suspended in cell culture media (RPMI 1640 containing 25 mM HEPES, 10 % FCS, 1 % NEAA, 5×10^–5^ M β-mercaptoethanol, 100 U penicillin ml^−1^ and 100 µg streptomycin ml^−1^) and added in duplicate (4×10^5^ cells per well) to the peptides in 96-well plates, which were then incubated at 37 °C in the presence of 5 % CO_2_ for three days, after which cell culture supernatants were harvested and stored at −80 °C until required. In both assays, pokeweed mitogen was included as a positive control at a final concentration of 10 µg ml^−1^, while RPMI 1640 alone served as a negative control. Quantification of IFN-γ in plasma or cell culture supernatant was performed using the Bovigam ELISA kit (Prionics). For the whole-blood assay, a result was considered positive if the OD_450_ with antigen minus the OD_450_ without antigen was ≥0.1, as previously reported (Jones *et al.*, 2010). For the PBMC assay, any OD_450_ value that was greater than a cut off calculated as the mean OD_450_ for 23 negative control wells plus three standard deviations plus 0.1 OD unit was considered positive.

## RESULTS

### Selection of alleles

Ideally the alleles selected as the basis for prediction should reflect the genetic diversity of the population under study, in this case that of European domestic cattle. However since the available prediction methods do not cover BoLA-DRB3 alleles we were required to use HLA alleles to approximate the bovine response; this restricted our coverage to a subset of BoLA-DRB3 alleles that are similar enough to HLA alleles to be accurate proxies. The population coverage is therefore partial and serves largely to remove alleles unlikely to be present in a bovine population. Although the DQ locus is also expressed and relevant to immune response in cattle, we did not consider it here as homologous human alleles are not covered by either of the predictors.

**Fig. 3.**
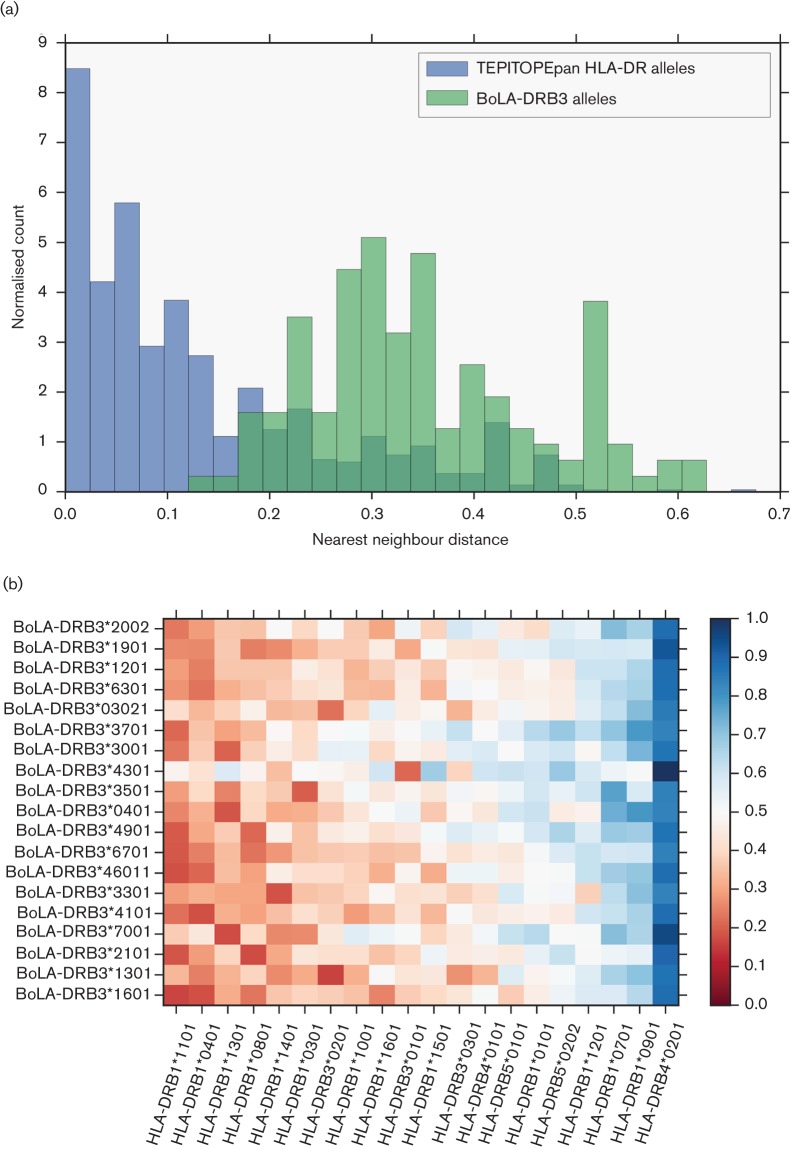
Similarity of human and bovine alleles. (a) Distributions of mean nearest neighbour distance to Tepitope library alleles for the 700 HLA alleles covered by TEPITOPEpan (blue) and known BoLA alleles (green). Though most of the BoLA pseudo-sequences are too distant to be used, there is a substantial overlapping region with the HLA alleles covered by TEPITOPEpan. (b) BoLA vs HLA alleles pseudo-sequence distances. The heatmap shows the nearest-neighbour distances between BoLA (y-axis) and closest HLA alleles covered by TEPITOPEpan. The colour scale represents pseudo-sequence distance on a scale of 0 to 1. Low values (red) are more closely matching alleles. The *x*-axis is sorted by mean distance. We choose the eight closest HLA alleles with nearest neighbour distance ≤0.25 (leftmost on the *x*-axis) for both our prediction methods.

The distributions of the average nearest neighbour (pseudo-sequence distance to the closest human allele) for all BoLA-DRB3 sequences and the set of 700 HLA-DR covered by Tepitope are compared in Fig. 3(a). The region of overlap illustrates that a subset of BoLA alleles are close enough to extrapolate predictions from human HLA-DR alleles to bovine BoLADRB3 binding peptides. We chose to use nearest distance ≤0.25 as a valid cut-off, based on predictor performance versus distance data from the literature (Nielsen *et al.*, 2008). We then used this cut-off to determine the set of HLA-DR alleles close enough on average to the BoLA sequences to be useful. Fig. 3(b) is a heat map showing the distances between the top closest BoLA-DRB3 and corresponding HLA-DR pseudo-sequences. There were eight representative HLA alleles close enough that cover a portion of the BoLA sequences, and these are listed in Table 2.

**Table 2.** Subset of HLA-DR alleles used for predictions

Reference	Nearest*	Mean
HLA-DRB1*0301	0.20	0.43
HLA-DRB1*0401	0.18	0.40
HLA-DRB1*0801	0.17	0.42
HLA-DRB1*1101	0.16	0.37
HLA-DRB1*1301	0.17	0.41
HLA-DRB1*1401	0.18	0.40
HLA-DRB3*0101	0.21	0.49
HLA-DRB3*0201	0.16	0.45

*Nearest-neighbour distance is the pseudo-sequence similarity to the closest BoLA allele. For efficiency one allele subtype is chosen to represent all alleles for that allotype, i.e. HLA-DRB1*0301 represents DRB1*03. HLA allele nomenclature is explained on the IMGT/HLA website (Robinson *et al.*, 2013).

For the MHC-I predictions we chose the first nine BoLA alleles that are available in the IEDB prediction tools using the ‘recommended’ method. These are BoLA-N:00101, BoLA-N:00201, BoLA-N:00301, BoLA-N:00401, BoLA-N:00501, BoLA-N:00601, BoLA-N:00801, BoLA-N:00901, and BoLA-N:01001.

### Bovine alleles in the target population

**Fig. 4.**
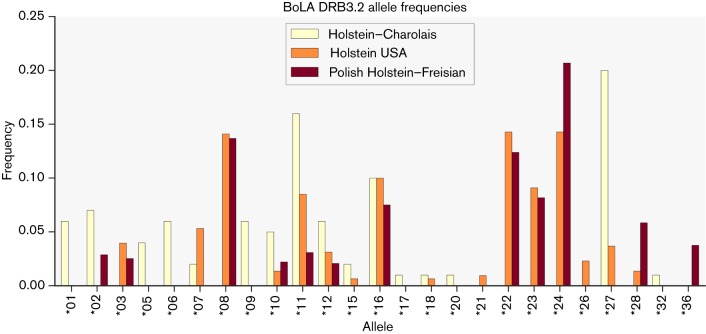
BoLA allele frequency distributions. Common allele frequencies for Holstein (USA) (Dietz *et al.*, 1997), Holstein–Charolais (UK) (Baxter *et al.*, 2008) and Polish Holstein–Friesian cattle (Oprządek *et al.*, 2012). Note that some frequency values represent the combination of two subtypes (e.g. *2703 and *2707). In these cases the higher value was used. Alleles with frequencies less than 2 % are not shown for clarity.

There is limited data on allelic frequencies for comparison but an investigation of the literature revealed several sources on allele representation in UK Holstein–Friesian herds. The first is a study of MHC genotyping in crossbred herd of 409 Holstein–Charolais cattle (Baxter *et al.*, 2008). This herd was found to have 24 distinct alleles with *2707 being the most frequent. The second is from a genotyping of 1100 Holstein cows from 93 dairy herds in Minnesota and Illinois (Dietz *et al.*, 1997). Finally a description of DRB3 polymorphism in 752 Polish Holstein–Friesian cattle (Oprządek *et al.*, 2012) in two distinct herds was used. All three datasets are compared in Fig. 4 showing good agreement where the studies overlapped in allele coverage. The Holstein–Charolais alleles can be seen to be more distinct, presumably due to the mixed nature of the herd. Several of the common BoLA alleles identified do indeed overlap with those covered by our HLA 8 alleles as indicated in Table 3 which shows frequencies for each allele across the three studies.

**Table 3.** Common BoLA-DRB3 allele frequencies

DRB3.2 alleles	Holstein–Charolais	Holstein (USA)	Polish Holstein–Freisian
*24	–	0.14	0.21
*08	–	0.14	0.14
*22^†^	–	0.14	0.12
*27	0.2	0.04	–
*11	0.16	0.09	0.03
*16^†^	0.1	0.10	0.08
*23	–	0.09	0.08
*06	0.06	–	–
*09	0.06	–	–
*01	0.06	–	–
*02	0.07	–	0.03
*05	0.04	–	–
*36^†^	–	–	0.04
*12^†^	0.06	0.03	0.03
*07	–	0.05	–
*28	–	–	0.06
*03	–	0.04	0.03
*10	0.05	–	0.02
*26	–	0.02	–

Shown are frequencies derived from USA Holstein (Dietz *et al.*, 1997), Holstein–Charolais (UK) (Baxter *et al.*, 2008) and Polish Holstein–Friesian cattle (Oprzadek *et al.*, 2012). ^†^Alleles covered by prediction methods.

### Peptide selection

We applied the two binding-prediction algorithms for all proteins in the *M. bovis* proteome. As described in the Methods the same eight HLA MHC-II alleles were used for both predictors and nine MHC-I BoLA alleles. This yielded large lists of binders from each prediction method. (For example there were 47 370 11-mer binders predicted by the TEPITOPEpan method). Any binders in proteins not meeting the absolute abundance and length criteria were removed from the analysis. This left 1871 proteins and 1272 after the length filter was applied. This gave final lists of 12 746 MHC-I, 7584 netMHCIIpan and 13 004 TEPITOPEpan binders respectively. All of these were 11-mer sequences.

The top-scoring binders selection strategy, was applied to both the MHC-II binder sets and resulted in 8834 shared binders found in 239 proteins. Filtering those with overlapping MHC-I binders produced a list of 454 peptide candidates; 42 of these overlapped with the binder clusters method and were removed. This final list was ranked by mean score.

For the binder clusters method, the cluster detection and overlap filters were applied to both sets of binders creating two sets of binder clusters. These were filtered and then ranked by binder density (binders per cluster). These two sets of 124 clusters and 131 clusters differ only in the order in which the binding predictions algorithms were applied.

### Experimental validation

For validation, 20-mer peptide sequences optimally covering the binders or cluster regions in each list were generated. A total of 376 peptides were synthesised as follows:

1. The 94 highest ranked top scoring binders.
2. The top 88 from each binder cluster set giving a total of 176.
3. A further random selection of 94 peptides covering low scoring binders constituted a ‘non-filtered’ control set.
4. 12 positive-control peptides representing known epitopes recognised by bovine CD4^+^ T cells from infected animals.

**Fig. 5.**
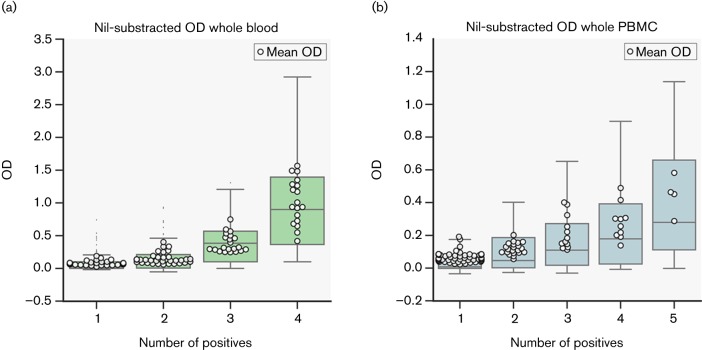
Results for both sets of IGRA assays. (a) IGRA whole-blood assays, in four animals. White data points are nil-subtracted mean OD values for all animals responding to a given peptide. The boxplots show the underlying distribution for the raw OD values (all animals). Results are grouped by each peptide's number of responders. Peptides inducing no responses are not shown. (b) PBMC IGRA assays in seven animals. White data points are nil-subtracted mean OD values with boxplots showing distribution for all data points.

All peptides were tested using whole blood (WB) (four animals) or PBMC (seven animals) collected from 11 field-reactor cattle naturally infected with *M. bovis.* Peptide-specific IFN-γ responses were determined by ELISA. Positive peptides were assigned as described in Methods. Mean OD values plotted by number of animals responding to peptide are shown in Fig. 5 for both sample types. When PBMC responses were considered, the maximal number of animals responding to a given peptide were five out of seven (Fig. 5b), whilst a number of peptides were recognised by four out of four animals providing whole blood for testing (Fig. 5a). Fig. 5 demonstrates that the IFN-γ responses towards individual peptides tended to increase with the degree of promiscuous recognition of individual peptides, i.e. peptides recognised by the five out of seven or four out of four cattle when tested as PBMC or whole-blood samples, respectively, induced the strongest IFN-γ responses. None of the 94 randomly selected control peptides induced whole-blood responses and only 2 out of 94 control peptides were recognised by two out of seven animals in the PBMC assay.

**Fig. 6.**
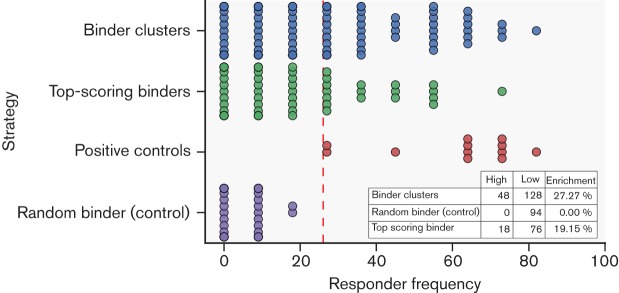
Enrichment of peptides containing epitopes predicted to bind to BoLA-DRB3. Responder frequencies (based on four whole-blood and seven PBMC samples) of all peptides tested were grouped by the epitope-selection strategy. Peptides were deemed promiscuous based on a cut-off of ≥26 % derived from the lowest positive control responder frequencies (purple). The cut-off level is indicated by the red dashed line. The binder clusters method should a superior enrichment of high response peptides at 27.3 %.

Whole-blood and PBMC responses of each peptide tested were integrated by adding the positives from both sample matrices. Responder frequency was then calculated as the fraction of positives out of the total 11 animals. Peptides were then ranked depending on responder frequency. The cut-off defining a high response was defined by the minimum responder frequencies for the positive control peptides, all of which are known to be promiscuously recognised. Thus, a cut-off of ≥26 % was used to define a high-responder or promiscuous peptide. Applying this interpretation, we defined 66 out of 270 peptides as being promiscuously recognised by bovine T cells (24.4 %), whilst none of the randomly selected control peptides met this criterion (Fig. 6). To extend this analysis, we tabulated these results broken down in relation to the two prediction strategies. Although there was no statistically significant difference between the success rate for predicting peptides recognised at high frequencies, the binder cluster method was marginally more successful (27.3 % compared with the top-binders method with 19.2 %). The randomly selected control set contained no peptides with a high response. Eleven peptides had responder frequencies greater than the mean response for the positive controls (60 %). These are listed in Table 4. The entire list of 376 peptides tested with responses for each animal and additional annotation is given in Table S1 (available in the online Supplementry Material).

**Table 4.** The 11 most frequently recognized peptides

***M. tuberculosis* H37Rv locus tag**	***M*. *bovis* tag**	Peptide sequence	Method	Responder frequency	*Start position
Rv3732	Mb3759	PYVRDGWAFVAIRLTSTDLI	Binder clusters	82	178
Rv1822	Mb1853	DWADGKIARLLNQSSRLGAL	Binder clusters	73	53
Rv2140c	Mb2164c	PGGALTLVNDAGMRRYVGAA	Top-scoring binders	73	102
Rv3671c	Mb3695c	NEAAPTWLKTVPKRLSALLN	Binder clusters	73	150
Rv3863	Mb3893	LAADGIINAGALIAFEKGRS	Binder clusters	73	183
Rv1239c	Mb1271c	PTVIGGMVLICLFLYHVFRN	Binder clusters	64	344
Rv1591	Mb1617	TQAPPVFFARRPLQIALTLM	Binder clusters	64	158
Rv1762c	Mb1793c	EHLEFMAVGTAVRYTAKPGA	Binder clusters	64	111
Rv1833c	Mb1864c	VMSSPPVQYAILRRNFFVER	Binder clusters	64	154
Rv2412	Mb2435	RNKAVKSSLRTAVRAFREAA	Binder clusters	64	20
Rv3247c	Mb3275c	ASSVYAMATLFALDRAGAVH	Binder clusters	64	62

Peptides with responder frequencies ≥60% of the mean value for the positive controls. *Start position is the location of the start of the peptide in the protein sequence. All peptides are 20 amino acids in length.

Next, we categorised the peptides into functional groups of the proteins they represent as defined at the TubercuList database, http://tuberculist.epfl.ch. There was no obvious enrichment of any functional categories by host protein of positive peptides compared with the negative set or the filtered genome as a whole.

### Conservation of predicted epitopes

We examined the conservation of positive and negative peptides across nine mycobacterial species by BLAST searching a local database and searching for the sequence substring within any orthologs. All epitopes were conserved between *M. bovis* and *M. tuberculosis* H37Rv and all but six are conserved with *Mycobacterium canetti*. The remaining species in the *Mycobacterium tuberculoisis* complex (MTBC) showed enough variation in conservation to allow an analysis of possible differences in the positive and negative set. We compared the proportions of peptides conserved for seven species between the two sets by performing a two-sample *Z*-test. No significant differences were seen using a *P*-value cut-off of 0.05.

## Discussion

Previous approaches for antigen identification in both *M. bovis* and *M. tuberculosis* have relied heavily on prior knowledge to guide selection, such as cellular location, functional classification or species distribution. Sources of suitable antigens are hence usually restricted to some known subset of proteins in the species. Few studies use purely computational methods relying on MHC binding predictions to screen and test epitopes on the scale we describe here. Our method is distinct in that it chooses from across the bacterial proteome, regardless of functional class. Our overall positive rate of 24.4 % for the predicted set of 270 epitopes shows the high success rate of the method, particularly when considered in the context of selection from 1 million peptides with no a priori knowledge of host proteins used apart from the abundance data.

It has been known for some time that secretion of antigenic proteins by mycobacteria induces strong cellular immune responses in the host. Members of the ESAT-6 protein family, for example, are among the most frequently recognized antigens from *M. bovis*. Our methods sample a different subset of proteins than those derived from such a knowledge-based perspective. A good illustration of this is provided by the well-studied bovine antigen Rv3874 (CFP-10). Promiscuous peptides recognized by *M. bovis*- infected cattle have previously been mapped by Vordermeier *et al.* (2001); comparing these mapped epitopes to the four promiscuous binders predicted by TEPITOPEpan shows that the predictions accurately reflect the epitopic sequence regions. However CFP-10 is not selected by our approach since it does not contain clusters and the binders are not highly ranked globally in terms of score. Several of our positives are contained in proteins previously cited in the literature as antigens, for example Rv1833c (Mb1864c) and Rv1239c (Mb1271c). However in general our predicted epitopes are found in proteins previously unexplored as antigens.

Since both of our epitope-enrichment strategies are essentially a set of iteratively applied filters, it is not possible to assert which steps are most effective in enrichment of positives. We applied sufficient filters to narrow down the search space to a reasonable number of testable peptides, i.e. the filters chosen were tailored to the target test set. However some general conclusions can be drawn from the results from each strategy. The cluster strategy had more success, with 27 % positives compared with an approximately 19 % positive rate for the top-binders strategy. Both performed far better than our set of randomised binders, which had no significant positives.

**Fig. 7.**
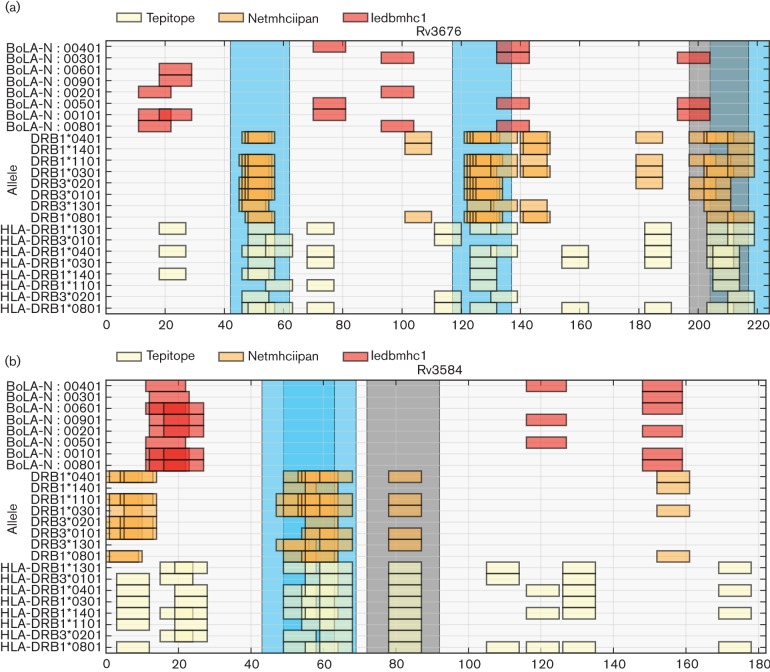
Sequences views of predicted binder clusters and positive epitope regions in selected proteins. (a) Rv3676 is the most enriched protein with three positive peptides out of four predicted. The peptide positions in the sequence are shown as regions in light blue with the single negative in grey. Each box marks the position of a predicted binder. Each row is one of the eight chosen HLA-DRB3 alleles (labelled on the y-axis) for each of three predictors. Colours represent each of the three predictors as shown in the legend. The plots were produced by the epitopepredict library. (b) Rv3854 had two positives from three predicted. The two positives (at positions 38 and 44) are overlapping and contain the same nine-mer core, LTINNVLLR.

Though the top-scoring binders strategy was also designed to find the proteins with highest number of epitopes, it did not enrich for epitopes in individual proteins with a few exceptions. Indeed it was observed that many of the positives from this method are also located within clusters of binders such as Rv3676 as shown in Fig. 7(a). Given these results we would recommend the binder cluster approach using both prediction algorithms as a general strategy. Binding promiscuity should also be a requirement but must be tailored to the target population and application. Other filters are likely to be less important and could be applied as needed to limit peptides for screening.

Our results are consistent with the findings of Weaver *et al.* (2008) who found that the immunodominance of a peptide tracks with the peptide itself, rather than the site in a given protein or the protein in which it is contained (though DM editing may also be vital). If this is the case, it is not clear why a peptide’s context apparently plays such a minor role in immunodominance. In this view binding affinity to MHC class II molecules of a peptide compared with its competitors is the overriding factor. Similar points have been made by Sette & Peters (2007), who point out that there can be no absolute correlation between antigen function and the ability of peptides to elicit immune responses. In the studies they reviewed it is seen that CDS from all functional categories, times of expression and cellular localizations are found to evoke responses. In this context, global selection from the proteome according to promiscuous binding, as we have attempted, is a rational approach.

For choice of binding-prediction algorithms we are for the moment constrained by availability of quality pan-specific methods. TEPITOPEpan was implemented because of its simplicity and the robustness of the PickPocket method (Zhang *et al.*, 2009) when the similarity to MHC molecules with known binding specificity is low. TEPITOPEpan [using the ProPred (Singh & Raghava, 2001) implementation] has already been used in several studies relating to *M. tuberculosis*, for example by Mustafa (2009). Our results show that this is still an effective algorithm when applied appropriately and was as successful as netMHCIIpan that is the current ‘state of the art’ algorithm.

It is very important to train binding-prediction methods on MHC binding data from the appropriate alleles. This data is usually obtained from MHC–peptide affinity competition assays. This is a relatively resource-intensive process that has to be done for each allele (Lundegaard *et al.*, 2012). For non-model organisms there is little such binding data. Even with trained predictors for some animal alleles, studies are typically limited by lack of information on the MHC haplotypes of the group under study. Without this information many more alleles than actually necessary might have to be covered. Performing predictions for all known alleles is only practical for screening a small number of known antigens or perhaps scanning a viral genome for epitopes (Liao *et al.*, 2013). The alternative is to make a best guess of the target alleles and select for promiscuity, as pursued here. MHC genotypes are rarely characterized comprehensively because of the prohibitive cost of standard technologies and the technical challenges of accurately discriminating between these highly related genes. With the advent of next-generation sequencing (Sommer *et al.*, 2013; Westbrook *et al.*, 2015) approaches this is due to change in the near future. The use of animal-allele-specific methods could create a significant improvement in accuracy for our method.

Our clusters method required at least one overlapping (within the 20-mer) predicted MHC-I binder to potentially enhance detection. For the top-scoring binders strategy, we did not apply this requirement (to avoid eliminating too many high scorers). We found that MHC class I binders frequently coincided with MHC-II binder clusters and single binders in any case. The randomised set of binders had 30 out of 94 with an overlapping binder and the top shared binder set had 46 out of 94. No correlation was observed between positive responses and MHC-I binder overlap. Also the number of overlapping MHC-I binders did not seem to affect the positive rate of peptides derived in the cluster strategy. It is probable that the effect of filtering for overlapping MHC-I binders is minimal though the number of data points is too small to make any conclusion. Our choice of MHC-I alleles was not optimal since we did not attempt to estimate the population-specific alleles and chose an arbitrary set. Data on bovine MHC-I haplotypes is available (Codner, 2010) and could be integrated into future predictions.

As noted already our predicted sequences had a bias for hydrophobic sequences, seen most obviously with the TEPITOPEpan method (Bian & Hammer, 2004). This is in part a fundamental property of the MHC ligand binding motif (Parry, 2008). Approximately 70 % of clusters found in the cluster strategy were inside or overlapped with transmembrane helix domains. Since this may have affected solubility we included a filter to remove strongly hydrophobic sequences using a relatively crude measure (simply counting the proportion of hydrophobic amino acid residues). However this could unnecessarily have removed actual positives and the omitted peptides may be tested in a later study. Hydrophobicity measures indicate no bias for negative or positive peptides.

Although the MHC-II processing pathway leading to CD4^+^ proliferation is still incompletely understood this knowledge could be used to narrow down the list of epitopes further in future predictions. For example, though the specificity of cathepsins (Zavasnik-Bergant & Turk, 2007) that control degradation of peptides is not well known, a method for cleavage prediction during peptide pre-processing has been developed by Hoze *et al.* (2013). However this tool is only available via a web page and was not practical for high-throughput use at the time the study was conducted.

The review by Lundegaard *et al.* (2012) has discussed the present status and future for computational epitope prediction systems. The authors stress that despite continued debate over the efficacy of prediction algorithms (Wu *et al.*, 2011) there is good reason to expect computational models to have a significant clinical effect in the coming years. This study has certainly reinforced the utility of such *in silico* methods, even using algorithms not specifically optimised for animal genotypes. In the veterinary field in particular, the rational design approach for vaccines and diagnostics has been very under-explored. This is partly because MHC genetic variation has been unmeasured (though it is now quite well known in cattle) and limited efforts have been made to derive binding data on the MHC alleles in question. This is likely to change in the future as MHC binding assays for animal species are developed.

### Conclusions

We have used two contrasting computational strategies based primarily on MHC-II binding predictions to select potential mycobacterial peptidic epitopes recognised by bovine T cells from the very large sequence space of the *M. bovis* proteome. Both of our methods were successful in capturing epitope-rich sequences using an almost purely computational approach, and our strategy based on finding regions of high-promiscuity binder clusters seems the most promising. These epitopes are excellent candidates for use in future studies of *M. bovis* diagnostics or potential sub-unit vaccines, while the computational methods presented here have general application in epitope selection for multiple infectious diseases.

## Supplementary Data

409 Supplementary File 1
