# Supplementary material for: Integrated computational prediction and experimental validation identifies promiscuous T cell epitopes in the proteome of Mycobacterium bovis
Source: Microb Genom. 2016 Aug 25;2(8):e000071. doi: 10.1099/mgen.0.000071 (PMC5320590; doi:10.1099/mgen.0.000071)
Supplement: Supplementary file 1 [file mgen-02-71-s001.pdf]

Appendix S1

Integrated computational prediction and experimental validation identifies promiscuous T cell epitopes in the proteome of *Mycobacterium bovis*.

Supplementary Information

Access to binding prediction results for individual proteins is best done via the web application Epitopemap.

Instructions:

- Navigate to http://enzyme.ucd.ie:8080/epitopemap
- Choose login from the top right menu
- Login as 'guest@ucd.ie' with password 'predict'
- Choose 'view predictions' from the main menu
- In the side panel choose id=results_emida, genome can be MTB-H37Rv or Mbovis. Predictions are specific to the 8 HLA alleles used for this study and some BoLA MHC-I alleles.
- You need to know the exact locus tag of the protein required. Enter it in the box and press update (or enter).
- A plot will appear and other tabs showing the binder data.
- If you don't know the locus tag of your gene you can go back to the main menu and choose 'find protein' and enter the keyword to search by. This searches the genbank file features.

Table S1: The entire list of 376 peptides tested with responses for each animal and additional annotation

	#pbmcpos	#wbpos	7010	7447	7448	7449	7852	7853	7855	8740	8742	8743	8744	R	RVL	binders	clustersize	concentration	end	gene	hydro	id	identity	iedbmhc1_11_overlap	iedbmhc1_9_overlap	locus_tag	mbname	method	mhc1overlaps	overlap	pep_no	peplength	peptide	score	start	tag	wbmean	confidence	strategy
140	0	0	0.127	0.053	0.14	0.0675	0.1295	0.073	0.059	0.0725	0.051	0.06	-0.0005					6	222		0.5	256		0	0	Rv2216	Mb2239	topsharedbinders	0		#107	20	EVRALQFAIAQPNLSGPVNL	0	202	Mb2239_256	0.04575	low	Top Scoring Binders
221	0	0	0.0685	0.078	0.189	0.073	0.141	0.102	0.06	0.0455	0.053	0.06	-0.007	4.472	0.35	2	20	1	235		0.45	55	100	5	3	Rv2410c	Mb2433c	cl_tepitopepan	8		#16	20	FMMLDRLFPRSVFHSLKLAE	0	215	Mb3269c_55	0.037875	low	Binder Clusters
119	0	3	0.072	0.053	0.05	0.089	0.0925	0.217	0.06	0.444	1.117	0.909	0.059	7.348	0.25	3	19	2	49		0.55	21	100	4	3	Rv3040c	Mb3066c	cl_tepitopepan	7		#258	20	SASGLAVFLMRRHAAMDFAA	27	30	Mb2000_21	0.63225	high	Binder Clusters
220	0	2	0.086	0.0535	0.083	0.0745	0.094	0.111	0.056	0.0545	0.3435	0.1615	0.0055	8.485	0.3	3	13	12	136	frr	0.3	16	100	1	0	Rv2882c	Mb2906c	cl_tepitopepan	1		#218	20	KGEEAKVSVRNIRRKAMEEL	18	123	Mb3259c_16	0.14125	low	Binder Clusters
121	0	0	0.127	0.0525	0.153	0.0675	0.0865	0.084	0.057	0.051	0.0515	0.0575	-0.003	2.449	0.3	2	19	1	86		0.35	175	99.7	2	0	Rv2017	Mb2040	cl_netmhciipan	2		#103	20	RFRGALAVDAHMRRHKTTKA	0	67	Mb2024_175	0.03925	low	Binder Clusters
122	0	0	0.0725	0.0545	0.13	0.071	0.084	0.104	0.056	0.0455	0.0805	0.082	-0.004	2.828	0.3	2	13	2	289	adhC	0.5	86	100	0	1	Rv3045	Mb3071	cl_tepitopepan	1		#262	20	PMAVPAFALALMRRSLAGSN	0	276	Mb2026c_86	0.051	low	Binder Clusters
123	0	0	0.164	0.0535	0.336	0.0745	0.141	0.097	0.058	0.091	0.0515	0.055	0.0005	5.477	0.35	3	20	2	185		0.55	44	100	2	0	Rv3218	Mb3244	cl_tepitopepan	2		#108	20	GGKVTAWRYIRAAVRAVLAC	0	165	Mb2080_44	0.0495	low	Binder Clusters
124	0	2	0.078	0.0515	0.116	0.0715	0.131	0.211	0.061	0.052	0.21	0.165	0.0055	5.292	0.3	2	18	7	66		0.3	46	100	0	1	Rv2061c	Mb2087c	cl_tepitopepan	1		#234	20	EKKSWKVKRIRNTPRVTLAT	18	48	Mb2087c_46	0.108125	low	Binder Clusters
219	0	2	0.0675	0.052	0.055	0.0755	0.121	0.124	0.069	0.106	0.233	0.5135	-0.004	5.477	0.35	6	20	1	113	truA	0.35	42	100	1	1	Rv3455c	Mb3484c	cl_tepitopepan	2		#77	20	ARFSALRRHYVYRLSTAPYG	18	93	Mb3244_42	0.212125	low	Binder Clusters
117	0	2	0.077	0.0535	0.076	0.069	0.094	0.091	0.067	0.0475	0.1645	0.2375	-0.0115	3.464	0.25	2	13	3	116	espD	0.55	77	100	1	0	Rv3614c	Mb3644c	cl_tepitopepan	1		#11	20	QLASEILVIADLARQKAQSA	18	103	Mb1914c_77	0.1095	low	Binder Clusters
218	0	3	0.053	0.052	0.053	0.0725	0.095	0.083	0.058	0.271	0.866	0.8765	0.0335	4.243	0.35	3	13	2	128	entC	0.55	58	100	1	0	Rv3215	Mb3241	cl_tepitopepan	1		#194	20	AFDGPLHKVVLARAVQLTAD	27	115	Mb3241_58	0.51175	high	Binder Clusters
217	0	0	0.085	0.0605	0.124	0.072	0.141	0.094	0.06	0.047	0.0605	0.0615	-0.004	7.483	0.35	2	12	7	186		0.4	20	99.6	0	1	Rv1978	Mb2000	cl_tepitopepan	1		#260	20	ATRVGKRFLIIDLKRQKPLS	0	174	Mb3228_20	0.04125	low	Binder Clusters
129	0	2	0.0665	0.0745	0.33	0.0705	0.175	0.124	0.056	0.053	0.3695	0.19	0.0025	5.292	0.35	2	17	2	257		0.45	47	100	2	2	Rv3218	Mb3244	cl_tepitopepan	4		#254	20	FATTSMKVVPTLRVVRQMFA	18	240	Mb2180c_47	0.15375	low	Binder Clusters
130	0	0	0.0935	0.0525	0.103	0.069	0.081	0.076	0.061	0.0495	0.053	0.0575	-0.002	2	0.3	2	19	1	113	dut	0.5	190	100	1	0	Rv2697c	Mb2716c	cl_netmhciipan	1		#145	20	LINLDPAAPIVVHRGDRIAQ	0	94	Mb2189c_190	0.0395	low	Binder Clusters
216	0	0	0.0995	0.0525	0.109	0.067	0.087	0.081	0.062	0.047	0.051	0.0555	-0.002	5.657	0.25	2	16	4	373		0.4	35	99.7	1	2	Rv3272	Mb3300	cl_tepitopepan	3		#146	20	RGADTITVIRTPARYASFRA	0	357	Mb3225c_35	0.037875	low	Binder Clusters
132	0	0	0.0735	0.066	0.107	0.0725	0.1275	0.088	0.06	0.0455	0.0505	0.062	-0.0125	2.828	0.35	2	17	2	128		0.35	93	100	0	1	Rv2915c	Mb2939c	cl_tepitopepan	1		#14	20	EDLPRIIRAGRHLARPKRYI	0	111	Mb2206c_93	0.036375	low	Binder Clusters
133	0	0	0.063	0.055	0.167	0.0825	0.1055	0.082	0.073	0.0535	0.0705	0.0825	-0.002	11.832	0.2	2	15	35	68	TB16.3	0.4	100	100	1	0	Rv2185c	Mb2207c	cl_netmhciipan	1		#53	20	RARMLMDAAIFKDTLIMSYE	0	53	Mb2207c_100	0.051125	low	Binder Clusters
134	0	0	0.065	0.066	0.155	0.09	0.104	0.109	0.06	0.0445	0.052	0.055	-0.016	2.449	0.35	2	14	1	143		0.35	176	100	1	1	Rv2190c	Mb2213c	cl_netmhciipan	2		#35	20	ESPQLLIDRLSVQRVMAHQM	0	129	Mb2213c_176	0.033875	low	Binder Clusters
135	0	0	0.0715	0.1185	0.104	0.0865	0.1235	0.083	0.064	0.048	0.058	0.0625	-0.004	12	0.15	2	18	24	31	inhA	0.5	3	100	0	1	Rv1484	Mb1520	cl_tepitopepan	1		#7	20	VSGIITDSSIAFHIARVAQE	0	13	Mb2225c_3	0.041125	low	Binder Clusters
127	0	0	0.116	0.052	0.091	0.069	0.1195	0.09	0.059	0.0935	0.0495	0.056	-0.001	6.481	0.25	3	19	7	82		0.4	27	99.6	0	1	Rv1978	Mb2000	cl_tepitopepan	1		#120	20	HEKYARIALKLVSDIPNPRI	0	63	Mb2174c_27	0.0495	low	Binder Clusters
116	0	0	0.1265	0.0545	0.137	0.0705	0.0915	0.084	0.056	0.061	0.0565	0.0565	-0.006	2	0.15	2	13	1	122	lipP	0.35	189	100	0	1	Rv2463	Mb2490	cl_netmhciipan	1		#124	20	GKQAITLAMVMSHRSGAIGP	0	109	Mb1911_189	0.042	low	Binder Clusters
115	0	0	0.0965	0.059	0.168	0.0715	0.1175	0.091	0.07	0.0715	0.0515	0.07	0.005					22	148		0.37	238		0	0	Rv1875	Mb1906	topsharedbinders	0		#131	19	AQEQRAVVLITPTRIYSNG	0	128	Mb1906_238	0.0495	low	Top Scoring Binders
114	0	3	0.105	0.055	0.133	0.075	0.1895	0.132	0.075	0.204	0.498	0.4615	0.0055					13	28		0.4	270		1	1	Rv1871c	Mb1902c	topsharedbinders	2		#271	20	REFVHRVQRFVVNPIGRQLP	27	8	Mb1902c_270	0.29225	high	Top Scoring Binders
186	0	0	0.0695	0.05	0.058	0.072	0.0915	0.07	0.059	0.058	0.0495	0.0515	-0.0115	3.873	0.3	3	18	1	184	tcrX	0.35	69	100	1	2	Rv3765c	Mb3791c	cl_tepitopepan	3		#46	20	EFELLRFMMHNSKRVLSKAQ	0	166	Mb2962_69	0.036875	low	Binder Clusters
94	0	1	0.068	0.0515	0.051	0.0735	0.089	0.076	0.055	0.0465	0.2355	0.1495	-0.003	6.708	0.35	3	14	3	37		0.45	112	100	0	1	Rv1598c	Mb1624c	cl_netmhciipan	1		#217	20	WLERLQVKYINRALKPIARY	9	23	Mb1624c_112	0.107125	low	Binder Clusters
95	0	0	0.056	0.0515	0.112	0.07	0.0875	0.069	0.056	0.0455	0.052	0.065	-0.004					10	75		0.3	214		0	0	Rv1626	Mb1652	topsharedbinders	0		#237	20	LHKPDLVIMDVKMPRRDGID	0	55	Mb1652_214	0.039625	low	Top Scoring Binders
96	0	0	0.069	0.0565	0.298	0.074	0.1215	0.085	0.058	0.056	0.0535	0.0605	-0.015					10	38		0.35	215		0	0	Rv1626	Mb1652	topsharedbinders	0		#63	20	AEDEALIRMDLAEMLREEGY	0	18	Mb1652_215	0.03875	low	Top Scoring Binders
232	0	0	0.094	0.0645	0.054	0.0775	0.1055	0.088	0.065	0.0655	0.0515	0.072	0.004	2.449	0.2	2	18	1	72		0.45	182	100	2	0	Rv3732	Mb3759	cl_netmhciipan	2		#152	20	ALLHWDGTTETIVMQLAMNA	0	54	Mb3348_182	0.04825	low	Binder Clusters
98	0	0	0.073	0.0505	0.28	0.0675	0.0925	0.08	0.058	0.0485	0.0565	0.062	-0.003					22	147		0.35	206		0	1	Rv1641	Mb1668	topsharedbinders	1		#216	20	SRPELGYRLLQRLGADVADY	0	127	Mb1668_206	0.041	low	Top Scoring Binders
99	0	0	0.0545	0.064	0.188	0.0695	0.111	0.072	0.057	0.0475	0.0575	0.103	-0.004					22	42		0.5	207		0	0	Rv1641	Mb1668	topsharedbinders	0		#195	20	GEQVGIVRIEDALRVAADAD	0	22	Mb1668_207	0.051	low	Top Scoring Binders
100	0	0	0.105	0.0515	0.066	0.068	0.193	0.071	0.056	0.071	0.05	0.0565	-0.0005	4	0.25	2	12	2	213		0.35	65	100	1	1	Rv2751	Mb2772	cl_tepitopepan	2		#101	20	AAPGSRMVFTYVRRDFIDGT	0	201	Mb1682_65	0.04425	low	Binder Clusters
101	0	0	0.068	0.0655	0.21	0.0775	0.09	0.117	0.06	0.055	0.058	0.069	-0.004	2.828	0.35	2	20	1	322		0.4	91	100	1	1	Rv2184c	Mb2206c	cl_tepitopepan	2		#244	20	SGSGLASIYRLRLALPQLDP	0	302	Mb1685_91	0.0445	low	Binder Clusters
102	0	0	0.179	0.078	0.209	0.1025	0.1205	0.083	0.061	0.1235	0.051	0.056	0.004	2.828	0.3	2	16	2	253		0.55	92	100	0	1	Rv2818c	Mb2842c	cl_tepitopepan	1		#114	20	RSATPAITIVLRAAVAKHLP	0	237	Mb1693_92	0.058625	low	Binder Clusters
231	0	0	0.067	0.0545	0.14	0.071	0.077	0.078	0.058	0.044	0.065	0.0555	-0.0095	3.464	0.35	2	12	1	73		0.4	80	100	3	2	Rv1828	Mb1859	cl_tepitopepan	5		#48	20	YDCARLRFILTAQRDHYLPL	0	61	Mb3342c_80	0.03875	low	Binder Clusters
228	0	0	0.206	0.053	0.275	0.0755	0.1275	0.12	0.057	0.148	0.061	0.0635	0	3.464	0.3	4	20	1	104	tmk	0.4	146	100	1	1	Rv3247c	Mb3275c	cl_netmhciipan	2		#119	20	CRGYDVVILDRYVASNAAYS	0	84	Mb3305_146	0.068125	low	Binder Clusters
227	0	0	0.051	0.0525	0.052	0.0675	0.0845	0.07	0.061	0.083	0.0675	0.0895	-0.0015	9.165	0.35	2	18	7	264	thrB	0.5	10	100	2	1	Rv1296	Mb1328	cl_tepitopepan	3		#149	20	AAMTASAEYLRLLRRHNVAA	0	246	Mb3302c_10	0.059625	low	Binder Clusters
225	0	3	0.0645	0.0745	0.227	0.0915	0.148	0.228	0.064	0.2005	0.5265	0.433	-0.0015	2.449	0.35	2	10	1	152	ephE	0.55	181	100	1	1	Rv3670	Mb3694	cl_netmhciipan	2		#57	20	ALLHSRLVRAIALISSPHPA	27	142	Mb3275c_181	0.289625	high	Binder Clusters
108	0	0	0.074	0.0605	0.211	0.084	0.1095	0.114	0.059	0.0465	0.058	0.0885	-0.003	9.592	0.15	2	18	23	182	sodA	0.5	7	99.5	0	1	Rv3846	Mb3876	cl_tepitopepan	1		#227	20	EHAFYLQYKNVKVDFAKAFW	0	164	Mb1858_7	0.0475	low	Binder Clusters
110	0	2	0.0625	0.05	0.097	0.1125	0.092	0.082	0.056	0.0515	0.2135	0.158	0.005	10.583	0.15	2	17	8	152		0.45	102	100	3	3	Rv1833c	Mb1864c	cl_netmhciipan	6		#270	20	TWFWPADTLAMKAFSRVMSS	18	135	Mb1864c_102	0.107	low	Binder Clusters
111	0	1	0.0705	0.0525	0.308	0.1165	0.1475	0.102	0.055	0.0475	0.2175	0.11	-0.0015	5.657	0.3	2	15	4	98	cysM	0.45	38	100	2	0	Rv1336	Mb1371	cl_tepitopepan	2		#238	20	NTGISLAMAARLKGYRLICV	9	83	Mb1864c_38	0.093375	low	Binder Clusters
223	0	0	0.0715	0.054	0.095	0.0735	0.082	0.074	0.061	0.0475	0.1485	0.118	-0.01					17	125		0.35	224		0	1	Rv3246c	Mb3274c	topsharedbinders	1		#2	20	PFKPKELVARVRARLRRNDD	0	105	Mb3274c_224	0.076	low	Top Scoring Binders
113	0	0	0.1	0.055	0.081	0.0735	0.096	0.095	0.065	0.0545	0.0485	0.0575	0.009	10.954	0.2	3	18	8	57		0.3	101	100	3	1	Rv1855c	Mb1886c	cl_netmhciipan	4		#143	20	DSLFVMDHFYQLPMLGTPDQ	0	39	Mb1886c_101	0.042375	low	Binder Clusters
214	0	0	0.0545	0.0505	0.132	0.0695	0.135	0.078	0.06	0.049	0.0935	0.0585	-0.013	6.928	0.33	3	21	4	350		0.38	111	99.7	2	0	Rv3168	Mb3193	cl_netmhciipan	2		#62	21	YVYSGVMWACVFMRTGARRVH	0	329	Mb3193_111	0.047	low	Binder Clusters
92	0	0	0.085	0.0535	0.055	0.071	0.078	0.077	0.06	0.142	0.072	0.0625	0.016	2.828	0.2	2	17	2	60	devB	0.4	88	100	1	0	Rv1445c	Mb1480c	cl_tepitopepan	1		#98	20	GGGNGIALLRYLSAQAQQIE	0	43	Mb1617_88	0.073125	low	Binder Clusters
213	0	2	0.049	0.05	0.054	0.0665	0.1185	0.072	0.058	0.046	0.2325	0.333	-0.0015	10	0.3	2	12	25	97	garA	0.3	6	100	1	0	Rv1827	Mb1858	cl_tepitopepan	1		#208	20	RHPDSDIFLDDVTVSRRHAE	18	85	Mb3186c_6	0.1525	low	Binder Clusters
139	0	0	0.093	0.0535	0.059	0.071	0.086	0.082	0.058	0.053	0.055	0.058	0.0035					6	186		0.35	255		0	3	Rv2216	Mb2239	topsharedbinders	3		#167	20	SPAGGMLRRMRPLFSVGLGA	0	166	Mb2239_255	0.042375	low	Top Scoring Binders
200	0	0	0.0815	0.088	0.329	0.076	0.1695	0.103	0.058	0.058	0.0535	0.056	-0.0065					8	222		0.47	273		0	0	Rv3000	Mb3025	topsharedbinders	0		#51	17	FDRAHRLVSLPAATRRA	0	202	Mb3025_273	0.04025	low	Top Scoring Binders
168	0	0	0.115	0.0515	0.064	0.0675	0.151	0.078	0.066	0.1035	0.08	0.077	0.012					7	304		0.63	268		0	1	Rv2579	Mb2610	topsharedbinders	1		#144	16	AAIAQFVRRLRSAAGV	0	284	Mb2610_268	0.068125	low	Top Scoring Binders
170	0	0	0.121	0.0555	0.088	0.069	0.0685	0.086	0.057	0.087	0.0795	0.1485	-0.0005	2.449	0.24	3	21	1	294	fadA5	0.43	172	100	0	1	Rv3546	Mb3576	cl_netmhciipan	1		#126	21	IVAQALVGAEPYYHLDGPVQS	0	273	Mb2615c_172	0.078625	low	Binder Clusters
197	0	2	0.086	0.0675	0.271	0.087	0.1475	0.113	0.068	0.0865	0.3995	0.7535	-0.001	18.974	0.35	2	12	90	103	leuB	0.4	1	99.7	0	1	Rv2995c	Mb3019c	cl_tepitopepan	1		#88	20	RFELDHHINLRPARLYPGVA	18	91	Mb3019c_1	0.309625	low	Binder Clusters
196	0	0	0.08	0.0535	0.105	0.0715	0.1325	0.072	0.064	0.0465	0.062	0.058	-0.0145	3.162	0.3	2	10	1	319	murX	0.3	153	100	2	2	Rv2156c	Mb2180c	cl_netmhciipan	4		#9	20	RTTGRRMFRMAPFHHHFELV	0	309	Mb3011c_153	0.038	low	Binder Clusters
195	0	1	0.073	0.148	0.083	0.0705	0.093	0.086	0.056	0.0455	0.307	0.056	-0.002	3.464	0.1	2	14	2	135	hsaG	0.35	152	100	1	1	Rv3535c	Mb3565c	cl_netmhciipan	1		#209	20	DAPNVNMITCGGQATIPIVY	9	121	Mb3011c_152	0.101625	low	Binder Clusters
194	0	0	0.085	0.0535	0.124	0.077	0.145	0.096	0.069	0.049	0.0495	0.0545	-0.014	7.071	0.35	2	18	5	346	lipN	0.45	109	100	2	2	Rv2970c	Mb2994c	cl_netmhciipan	4		#13	20	KALRAAGTAVDLRYLGSLTH	0	328	Mb2994c_109	0.03475	low	Binder Clusters
175	0	2	0.075	0.0525	0.313	0.0745	0.111	0.104	0.061	0.088	0.4145	0.325	-0.007	8.944	0.25	2	16	10	30	ahpD	0.35	13	100	1	1	Rv2429	Mb2455	cl_tepitopepan	2		#3	20	EYAKDIKLNLSSITRSSVLD	18	14	Mb2734_13	0.205125	low	Binder Clusters
201	0	0	0.0665	0.056	0.087	0.0775	0.233	0.117	0.064	0.0475	0.105	0.0755	0.0005	5.292	0.25	2	12	2	47		0.55	128	100	3	3	Rv3040c	Mb3066c	cl_netmhciipan	6		#40	20	ASGLAVFLMRRHAAMDFAAG	0	35	Mb3066c_128	0.057125	low	Binder Clusters
192	0	1	0.0755	0.054	0.112	0.07	0.114	0.143	0.058	0.104	0.337	0.141	-0.0205	7.071	0.3	2	17	5	146		0.5	108	99.7	2	2	Rv2955c	Mb2979c	cl_netmhciipan	4		#275	20	DRCILDVGANVGIHSLAWAR	9	129	Mb2979c_108	0.140375	low	Binder Clusters
178	0	0	0.147	0.054	0.063	0.076	0.0865	0.084	0.06	0.054	0.0515	0.0555	0.008	3.873	0.19	3	21	1	99		0.52	145	100	2	1	Rv3277	Mb3305	cl_netmhciipan	3		#132	21	IVGGTTFIIDTAIFYTLKLTV	0	78	Mb2809c_145	0.04225	low	Binder Clusters
189	0	0	0.071	0.0705	0.21	0.0755	0.1805	0.088	0.062	0.0455	0.0505	0.0575	-0.0145	6.481	0.25	3	20	7	42		0.4	114	100	1	1	Rv2952	Mb2976	cl_netmhciipan	1		#37	20	WRYWYPLMTRGLGNDEIVFI	0	22	Mb2976_114	0.03475	low	Binder Clusters
180	0	0	0.1025	0.0565	0.149	0.0665	0.0885	0.08	0.06	0.057	0.0535	0.0575	0.003					23	112		0.5	195		1	1	Rv2803	Mb2826	topsharedbinders	2		#148	20	GQSLSGFVLSAAVDHAHDLL	0	92	Mb2826_195	0.04275	low	Top Scoring Binders
188	0	2	0.0585	0.05	0.14	0.075	0.1035	0.115	0.061	0.0805	0.407	0.2185	0.0025	9.381	0.15	2	12	22	301	fadE25	0.5	9	100	1	0	Rv3274c	Mb3302c	cl_tepitopepan	1		#266	20	STFQAVQFMLADMAMKVEAA	18	289	Mb2973c_9	0.177125	low	Binder Clusters
182	0	1	0.054	0.0695	0.051	0.122	0.0745	0.102	0.062	0.052	0.1485	0.1635	-0.0145	3.464	0.25	2	15	3	60	leuD	0.45	151	100	1	0	Rv2987c	Mb3011c	cl_netmhciipan	1		#27	20	FAGWRSDPAFVLNLSPFDRG	9	45	Mb2898_151	0.087375	low	Binder Clusters
183	0	1	0.1245	0.052	0.113	0.069	0.139	0.095	0.065	0.052	0.517	0.108	-0.003	5.477	0.15	2	17	5	159	mpt53	0.35	124	100	1	1	Rv2878c	Mb2903c	cl_netmhciipan	2		#189	20	VFYRADGTSTFVNNPTAAMS	9	142	Mb2903c_124	0.1685	low	Binder Clusters
184	0	0	0.098	0.055	0.162	0.0785	0.124	0.102	0.058	0.0455	0.114	0.083	0.0015	2.828	0.35	2	19	1	79		0.55	95	99.7	1	0	Rv3092c	Mb3119c	cl_tepitopepan	1		#231	20	ELPIIKRIAIGSVRNKLLLI	0	60	Mb2939c_95	0.061	low	Binder Clusters
185	0	0	0.071	0.078	0.253	0.073	0.17	0.086	0.059	0.0435	0.049	0.056	-0.0105	3.464	0.3	2	16	3	268		0.4	148	100	0	1	Rv1245c	Mb1277c	cl_netmhciipan	1		#39	20	DLVVRLTGSGYQRIFPIITG	0	252	Mb2962_148	0.0345	low	Binder Clusters
177	0	1	0.0785	0.0535	0.149	0.068	0.1325	0.084	0.062	0.0505	0.4145	0.0585	-0.0055	6	0.29	3	21	6	33		0.48	119	100	1	1	Rv2778c	Mb2800c	cl_netmhciipan	1		#190	21	VEIDANPDLVYGLITDLPTLA	9	12	Mb2800c_119	0.1295	low	Binder Clusters
202	0	0	0.079	0.122	0.141	0.17	0.192	0.121	0.062	0.0505	0.0535	0.0885	-0.015	6.928	0.35	2	19	6	158		0.5	22	100	2	0	Rv2454c	Mb2481c	cl_tepitopepan	2		#20	20	GGNHLIHALRRNINVTILLF	0	139	Mb3066c_22	0.044375	low	Binder Clusters
164	0	0	0.067	0.053	0.053	0.072	0.122	0.095	0.064	0.0465	0.11	0.0625	-0.011	2.828	0.35	2	12	1	83	vapC17	0.35	165	100	1	1	Rv2527	Mb2556	cl_netmhciipan	2		#4	20	DIFDRVRHLQRDLAHHRGMW	0	71	Mb2556_165	0.052	low	Binder Clusters
163	0	0	0.0995	0.053	0.052	0.076	0.0855	0.076	0.059	0.069	0.058	0.1135	0.0005	3	0.35	3	17	1	108		0.55	157	100	0	2	Rv2313c	Mb2340c	cl_netmhciipan	2		#169	20	GAVVAMYIADFVPRVRAGLE	0	91	Mb2545c_157	0.06025	low	Binder Clusters
280	0	0	0.1145	0.051	0.102	0.0695	0.1175	0.087	0.061	0.0965	0.0605	0.143	0.0045	3.873	0.3	3	19	1	105	ribF	0.5	144	100	2	2	Rv2786c	Mb2809c	cl_netmhciipan	4		#182	20	ELVQDLGIEVFLVMPFTTDF	0	86	Mb3952c_144	0.076125	low	Binder Clusters
211	0	0	0.081	0.0515	0.241	0.073	0.124	0.1	0.066	0.045	0.1065	0.0615	0.0035	5.477	0.3	2	12	3	146	nuoC	0.25	126	100	1	2	Rv3147	Mb3171	cl_netmhciipan	3		#226	20	ETGRELHAVYPLQSITHNRR	0	134	Mb3171_126	0.054125	low	Binder Clusters
142	0	2	0.0515	0.0515	0.05	0.0695	0.0695	0.074	0.056	0.221	0.0745	0.179	0.025	2.828	0.35	2	11	1	182		0.35	163	100	1	1	Rv2252	Mb2276	cl_netmhciipan	3		#170	20	WPHGRMRYYIAMLAELSRLR	18	171	Mb2276_163	0.124875	low	Binder Clusters
143	0	0	0.047	0.049	0.051	0.0665	0.077	0.07	0.055	0.0615	0.123	0.118	0.01	3	0.35	3	18	1	201		0.4	156	99.7	1	1	Rv1708	Mb1735	cl_netmhciipan	2		#207	20	YDYVLIDCQPSLGLLTVNGL	0	183	Mb2307c_156	0.078125	low	Binder Clusters
145	0	1	0.0835	0.053	0.053	0.067	0.106	0.078	0.057	0.137	0.103	0.537	0.0115	4.899	0.3	2	12	6	101		0.5	49	100	1	0	Rv3094c	Mb3121c	cl_tepitopepan	1		#171	20	AITSALLVAYLPARSREEMF	9	89	Mb2311_49	0.197125	low	Binder Clusters
146	0	0	0.0725	0.05	0.075	0.07	0.088	0.093	0.056	0.045	0.051	0.0595	-0.006	12.728	0.15	2	16	27	73		0.3	2	100	1	1	Rv2296	Mb2318	cl_tepitopepan	2		#253	20	EPTWSYLYRTMIPPLSAAGH	0	57	Mb2318_2	0.037375	low	Binder Clusters
147	0	0	0.0895	0.0555	0.277	0.0855	0.1275	0.112	0.064	0.0445	0.051	0.06	-0.011	6.481	0.3	2	16	3	293		0.45	30	100	2	3	Rv3680	Mb3705	cl_tepitopepan	5		#42	20	MPLAGLVFNRTHPMLCALPI	0	277	Mb2320_30	0.036125	low	Binder Clusters
148	0	0	0.083	0.0745	0.091	0.114	0.097	0.103	0.061	0.0455	0.05	0.0765	-0.014					9	51		0.3	282		1	1	Rv2302	Mb2324	topsharedbinders	2		#17	20	DGSPPYVVRWLVNGHETTVY	0	31	Mb2324_282	0.0395	low	Top Scoring Binders
149	0	0	0.089	0.054	0.099	0.074	0.148	0.102	0.063	0.082	0.0805	0.116	0.011	3	0.29	3	21	1	98	folE	0.43	159	100	2	0	Rv3609c	Mb3639c	cl_netmhciipan	2		#130	21	LVLVKEIPMYSTCEHHLVAFH	0	77	Mb2340c_159	0.072375	low	Binder Clusters
150	0	1	0.069	0.12	0.34	0.085	0.1685	0.137	0.058	0.1315	0.2115	0.061	0.003	2.828	0.25	2	19	2	255	phoH1	0.35	164	100	0	1	Rv2368c	Mb2389c	cl_netmhciipan	1		#74	20	YMRGRTLNDAFIVLDEAQNT	9	236	Mb2389c_164	0.10175	low	Binder Clusters
151	0	0	0.0845	0.0535	0.071	0.1105	0.069	0.095	0.057	0.0475	0.052	0.054	-0.0145	4.472	0.3	4	20	1	48		0.5	56	100	3	1	Rv3651	Mb3675	cl_tepitopepan	4		#12	20	PITTFLRRSPYLAAVRTAIA	0	28	Mb2433c_56	0.03475	low	Binder Clusters
153	0	0	0.068	0.0585	0.215	0.0705	0.135	0.11	0.067	0.0455	0.0595	0.0585	-0.001	4	0.25	2	19	2	31		0.4	66	100	1	0	Rv3198A	Mb3223	cl_tepitopepan	1		#205	20	SWCGYCLRLKTALTANRIAY	0	12	Mb2440c_66	0.040625	low	Binder Clusters
154	0	2	0.065	0.079	0.128	0.099	0.207	0.15	0.059	0.048	0.189	0.1695	-0.015	2.828	0.35	2	18	2	180		0.5	84	100	0	0	Rv2003c	Mb2026c	cl_tepitopepan	1		#19	20	TLCFVTDPAAIFRETRRLLA	18	162	Mb2450c_84	0.097875	low	Binder Clusters
208	0	0	0.064	0.05	0.187	0.0675	0.0755	0.087	0.058	0.045	0.0495	0.063	-0.0075					6	137		0.56	274		0	0	Rv3143	Mb3167	topsharedbinders	0		#245	16	IVLGRTVLSLLRAPAH	0	117	Mb3167_274	0.0375	low	Top Scoring Binders
156	0	0	0.089	0.0535	0.167	0.0885	0.1085	0.122	0.063	0.056	0.0485	0.056	0.0025	8.718	0.15	2	18	19	172		0.15	14	100	1	1	Rv1703c	Mb1729c	cl_tepitopepan	1		#160	20	PGAPKYRAYMRRQQGMSWNT	0	154	Mb2455_14	0.04075	low	Binder Clusters
205	0	0	0.073	0.05	0.311	0.076	0.0975	0.088	0.059	0.0465	0.052	0.053	-0.013	4.899	0.3	4	19	2	200		0.45	53		1	1	Rv1516c		cl_tepitopepan	2		#47	20	DVMPLDWYLHVRHAVHGDIA	0	181	Mb3121c_53	0.034625	low	Binder Clusters
160	0	0	0.084	0.0525	0.079	0.07	0.0935	0.078	0.062	0.067	0.0515	0.057	0.0115	6	0.35	2	16	3	86	glbO	0.35	121	100	2	3	Rv2470	Mb2497	cl_netmhciipan	5		#138	20	HPRLRMRHAPFRISLIERDA	0	70	Mb2497_121	0.04675	low	Binder Clusters
161	0	0	0.233	0.062	0.257	0.0755	0.136	0.074	0.06	0.1105	0.06	0.0815	0.011	4	0.25	2	17	2	40		0.3	67	99.7	1	1	Rv1367c	Mb1402c	cl_tepitopepan	2		#133	20	GVPMQRDTLFRIASMTKPVT	0	23	Mb2528c_67	0.06575	low	Binder Clusters
203	0	1	0.055	0.0505	0.069	0.077	0.089	0.087	0.062	0.088	0.248	0.119	-0.011	2.828	0.25	2	14	2	197	cmaA1	0.5	87	99.7	0	1	Rv3392c	Mb3424c	cl_tepitopepan	1		#70	20	RGLPMSFTFARFLKFIVTEI	9	183	Mb3071_87	0.111	low	Binder Clusters
138	0	2	0.1045	0.0545	0.129	0.076	0.1055	0.097	0.057	0.099	0.185	0.274	0.0045					6	44		0.45	254		0	0	Rv2216	Mb2239	topsharedbinders	0		#95	20	AADHTVLRIVRRAPANSEEL	18	24	Mb2239_254	0.140625	low	Top Scoring Binders
90	0	0	0.0785	0.125	0.098	0.0985	0.147	0.108	0.06	0.0465	0.0505	0.0555	-0.0145					51	94		0.45	235		0	0	Rv1544	Mb1571	topsharedbinders	0		#25	20	AQLADRLRSDAIAGLCNSAG	0	74	Mb1571_235	0.0345	low	Top Scoring Binders
233	0	0	0.1085	0.051	0.053	0.0655	0.0695	0.073	0.054	0.0805	0.0525	0.0585	0.0005	6	0.3	3	20	3	31		0.3	33	100	2	2	Rv2954c	Mb2978c	cl_tepitopepan	2		#102	20	ERHFHSIFYLRHNARRQEHL	0	11	Mb3391c_33	0.048	low	Binder Clusters
88	0	0	0.121	0.052	0.128	0.071	0.103	0.092	0.06	0.0655	0.0525	0.0565	0.007	6.633	0.25	2	14	11	278		0.4	113	100	1	0	Rv1543	Mb1570	cl_netmhciipan	1		#106	20	QAAGVITDAIVHRPRRASSP	0	264	Mb1570_113	0.045375	low	Binder Clusters
27	0	0	0.0915	0.074	0.165	0.071	0.103	0.099	0.058	0.049	0.0525	0.0695	0.0015					15	152		0.35	226		1	1	Rv0464c	Mb0473c	topsharedbinders	2		#228	20	LQATDELIKDRTITAGTWER	0	132	Mb0473c_226	0.043125	low	Top Scoring Binders
28	0	0	0.065	0.0545	0.052	0.0675	0.2345	0.073	0.06	0.0735	0.081	0.0665	0.003					8	253		0.45	278		0	1	Rv0500	Mb0511	topsharedbinders	1		#150	20	NGELMGLRVDLTASRLRAAV	0	233	Mb0511_278	0.056	low	Top Scoring Binders
29	0	2	0.077	0.1165	0.302	0.1125	0.161	0.153	0.063	0.0525	0.2375	0.183	-0.01					8	135		0.55	279		1	1	Rv0500	Mb0511	topsharedbinders	1		#26	20	LPAGTPVVRAMPNAAALVGA	18	115	Mb0511_279	0.11575	low	Top Scoring Binders
257	0	0	0.0715	0.05	0.051	0.071	0.079	0.069	0.056	0.069	0.053	0.0815	0.0045	4.243	0.3	2	16	3	126		0.45	57	100	1	1	Rv2954c	Mb2978c	cl_tepitopepan	2		#99	20	VYCYGVLYHLSRPAEALAWM	0	110	Mb3675_57	0.052	low	Binder Clusters
31	0	0	0.0525	0.055	0.057	0.078	0.1635	0.08	0.057	0.047	0.0565	0.064	-0.006					26	114		0.45	196		0	0	Rv0580c	Mb0595c	topsharedbinders	0		#243	20	TAMRGELIRDRAVVSELFLR	0	94	Mb0595c_196	0.040375	low	Top Scoring Binders
32	0	1	0.1165	0.0525	0.053	0.0775	0.0915	0.075	0.059	0.048	0.0505	0.056	0.535					26	31		0.5	197		2	1	Rv0580c	Mb0595c	topsharedbinders	3		#125	20	HPPAALLRLVNPILRSLLHT	9	11	Mb0595c_197	0.172375	low	Top Scoring Binders
33	0	0	0.0825	0.0565	0.055	0.07	0.091	0.082	0.057	0.0495	0.052	0.0655	-0.0065					39	97		0.3	217		1	0	Rv0655	Mb0674	topsharedbinders	1		#247	20	RPERGSIIDGTDIIECSAK	0	77	Mb0674_217	0.040125	low	Top Scoring Binders
34	0	1	0.085	0.056	0.321	0.117	0.112	0.079	0.059	0.0515	0.1575	0.06	-0.0115					39	191		0.5	218		0	0	Rv0655	Mb0674	topsharedbinders	0		#64	20	AGLARALVLDPQIILCDEPD	9	171	Mb0674_218	0.064375	low	Top Scoring Binders
258	0	1	0.15	0.052	0.064	0.0705	0.0885	0.077	0.057	0.7875	0.0565	0.064	0.0055	2	0.35	2	10	1	195		0.5	184	100	1	0	Rv1289	Mb1320	cl_netmhciipan	1		#127	20	QDHFREIVNVDRASLVALDF	9	185	Mb3694_184	0.228375	low	Binder Clusters
35	0	0	0.0505	0.0535	0.053	0.084	0.068	0.079	0.06	0.0455	0.052	0.054	-0.014					39	218		0.5	219		1	1	Rv0655	Mb0674	topsharedbinders	1		#28	20	TAYLSQLIMDINAQIDATIL	0	198	Mb0674_219	0.034375	low	Top Scoring Binders
255	0	0	0.086	0.052	0.094	0.068	0.091	0.083	0.062	0.053	0.0645	0.0595	0.0005	2.449	0.35	2	11	1	88		0.45	183	100	1	1	Rv3814c	Mb3844c	cl_netmhciipan	2		#214	20	QDVRAVNYVIKHAQLIPVDR	0	77	Mb3639c_183	0.044375	low	Binder Clusters
253	0	0	0.1395	0.0755	0.187	0.068	0.159	0.085	0.062	0.0805	0.0725	0.0775	0	2.828	0.25	2	11	2	171	canB	0.45	168	99.5	0	1	Rv3588c	Mb3619c	cl_netmhciipan	1		#111	20	HVHETVAILMARSSAISERI	0	160	Mb3619c_168	0.057625	low	Binder Clusters
39	0	0	0.166	0.0515	0.054	0.0685	0.085	0.077	0.06	0.055	0.049	0.0585	0.005					17	101		0.45	192		0	0	Rv0700	Mb0720	topsharedbinders	0		#147	20	KTVDALMRIDLPASVDVNIQ	0	81	Mb0720_192	0.041875	low	Top Scoring Binders
40	0	0	0.125	0.052	0.053	0.071	0.078	0.074	0.056	0.0555	0.053	0.0585	-0.0025					17	63		0.15	193		0	0	Rv0700	Mb0720	topsharedbinders	0		#109	20	TEKNVYCVIRSPHKYKDSRE	0	43	Mb0720_193	0.041125	low	Top Scoring Binders
41	0	0	0.2075	0.0525	0.135	0.071	0.144	0.115	0.058	0.095	0.064	0.1105	0.0045					13	208		0.5	266		0	0	Rv0702	Mb0722	topsharedbinders	0		#121	20	LNTYDVLRADDVVFSVEALN	0	188	Mb0722_266	0.0685	low	Top Scoring Binders
42	0	0	0.079	0.0825	0.097	0.1385	0.1535	0.178	0.066	0.0485	0.14	0.0785	-0.0125					13	22		0.3	267		0	0	Rv0702	Mb0722	topsharedbinders	0		#22	20	AQEQKTLKIDVKTPAGKVDG	0	2	Mb0722_267	0.063625	low	Top Scoring Binders
89	0	0	0.083	0.0685	0.064	0.0825	0.1075	0.09	0.062	0.045	0.0545	0.0595	-0.0065					51	268		0.37	234		1	1	Rv1544	Mb1571	topsharedbinders	1		#6	19	SLLLPAIRIGNRLRGGPSR	0	248	Mb1571_234	0.038125	low	Top Scoring Binders
44	0	0	0.138	0.052	0.09	0.069	0.095	0.08	0.059	0.0565	0.049	0.0535	0					9	51		0.45	286		1	0	Rv0707	Mb0727	topsharedbinders	1		#112	20	VKEDVAIRRLLSSGLERAGI	0	31	Mb0727_286	0.03975	low	Top Scoring Binders
256	0	1	0.0705	0.0535	0.176	0.072	0.109	0.104	0.06	0.055	0.2225	0.1505	-0.006	3.464	0.2	2	20	2	90		0.45	78	100	1	1	Rv3575c	Mb3606c	cl_tepitopepan	2		#75	20	GLVMAEPLTYFFSDPAARDF	9	70	Mb3644c_78	0.1055	low	Binder Clusters
25	0	0	0.1835	0.052	0.052	0.0695	0.2365	0.075	0.058	0.128	0.0525	0.063	0.015					8	33		0.4	258		0	1	Rv0250c	Mb0256c	topsharedbinders	1		#115	20	VGGLRRCVTALKARFGDNPA	0	13	Mb0256c_258	0.064625	low	Top Scoring Binders
24	0	1	0.075	0.0615	0.137	0.0715	0.164	0.091	0.067	0.0725	0.1995	0.1145	-0.003					18	84		0.15	242		1	0	Rv0247c	Mb0253c	topsharedbinders	1		#83	20	INGKPRLMCMTRMSTFDEDE	9	64	Mb0253c_242	0.095875	low	Top Scoring Binders
23	0	0	0.0915	0.1045	0.064	0.07	0.1015	0.076	0.061	0.053	0.049	0.061	0.0055					18	98		0.35	241		1	0	Rv0247c	Mb0253c	topsharedbinders	1		#151	20	TFDEDEIVTVTPMRTFPVIR	0	78	Mb0253c_241	0.042125	low	Top Scoring Binders
278	0	0	0.074	0.06	0.183	0.0835	0.1765	0.086	0.059	0.0495	0.0575	0.0665	-0.0105	4	0.3	2	17	2	136		0.5	139	99.4	1	2	Rv2616	Mb2649	cl_netmhciipan	3		#81	20	FAVRCDPMTSVVFAEVLSFS	0	119	Mb3948c_139	0.04075	low	Binder Clusters
275	0	2	0.0625	0.0535	0.225	0.101	0.22	0.16	0.065	0.0645	0.303	0.217	-0.0015	2.828	0.3	2	14	1	206		0.45	170	100	2	1	Rv3856c	Mb3886c	cl_netmhciipan	3		#267	20	DRLDIVVASVHSKLSMDSAA	18	192	Mb3886c_170	0.14575	low	Binder Clusters
273	0	0	0.0735	0.076	0.272	0.093	0.0975	0.088	0.057	0.0465	0.056	0.0655	-0.011	2	0.3	2	10	1	257		0.35	186	100	1	0	Rv1879	Mb1911	cl_netmhciipan	1		#65	20	LHKANPLYLLDFLRQSGNTP	0	247	Mb3844c_186	0.03925	low	Binder Clusters
272	0	0	0.054	0.052	0.07	0.0675	0.076	0.072	0.062	0.0465	0.1125	0.14	0.0055	9.165	0.35	4	14	3	249	dprE2	0.45	105	100	3	1	Rv3791	Mb3820	cl_netmhciipan	4		#233	20	AAFRYVMMVLRHIPRSIFRK	0	235	Mb3820_105	0.076125	low	Binder Clusters
271	0	2	0.0655	0.0525	0.052	0.073	0.0765	0.072	0.062	0.056	0.4345	0.247	-0.0105	3.464	0.3	3	18	2	273		0.4	71	100	0	1	Rv1882c	Mb1914c	cl_tepitopepan	1		#5	20	KGVSPEFVRRHIAKSLATLE	18	255	Mb3791c_71	0.18175	low	Binder Clusters
268	0	2	0.1665	0.051	0.052	0.068	0.0835	0.081	0.057	0.169	0.14	0.1655	0.0145	5.657	0.3	4	17	2	209	ligC	0.35	39	99.7	0	1	Rv3731	Mb3758	cl_tepitopepan	1		#105	20	QPDKRVMFKIKHLRTADCVV	18	192	Mb3758_39	0.12225	low	Binder Clusters
267	0	0	0.1395	0.0585	0.153	0.069	0.0865	0.082	0.056	0.0495	0.0525	0.054	0.002	3.464	0.3	2	18	1	89		0.45	76	100	0	4	Rv3717	Mb3744	cl_tepitopepan	4		#104	20	EHTFTWETGLRLRAALNALG	0	71	Mb3744_76	0.0395	low	Binder Clusters
266	0	0	0.057	0.0525	0.059	0.0725	0.163	0.075	0.06	0.0505	0.0515	0.0595	0.0035	6.481	0.35	2	16	7	53		0.5	29	100	1	1	Rv2298	Mb2320	cl_tepitopepan	2		#168	20	TGAARDIVKRARALGVTLFD	0	37	Mb3735c_29	0.04125	low	Binder Clusters
263	0	3	0.075	0.084	0.308	0.1495	0.1015	0.164	0.062	0.151	0.5575	0.6025	0.0035					21	224		0.35	201		0	0	Rv3676	Mb3700	topsharedbinders	0		#59	20	RLEGKSVLISDSERLARRAR	27	204	Mb3700_201	0.328625	high	Top Scoring Binders
12	0	0	0.0785	0.0795	0.296	0.129	0.0995	0.094	0.061	0.044	0.0735	0.058	-0.0145					45	98		0.28	243		0	0	Rv0053	Mb0054	topsharedbinders	0		#21	18	LSLNESVLRTKVMRTDKH	0	78	Mb0054_243	0.04025	low	Top Scoring Binders
13	0	0	0.066	0.0575	0.137	0.112	0.102	0.087	0.059	0.047	0.0555	0.0575	-0.0055					45	73		0.5	244		0	1	Rv0053	Mb0054	topsharedbinders	1		#52	20	KHAEGIYVVIDVKAAPATVS	0	53	Mb0054_244	0.038625	low	Top Scoring Binders
14	0	0	0.071	0.0555	0.059	0.075	0.103	0.072	0.07	0.047	0.062	0.0555	-0.007					22	129		0.35	237		0	0	Rv0056	Mb0057	topsharedbinders	0		#1	20	PNLDKRIVRLPKTHIKAVGT	0	109	Mb0057_237	0.039375	low	Top Scoring Binders
15	0	0	0.089	0.055	0.247	0.0705	0.1385	0.088	0.058	0.05	0.05	0.052	-0.013					11	175		0.45	276		0	0	Rv0146	Mb0151	topsharedbinders	0		#10	20	REVPADLRQDWPAALRDAGF	0	155	Mb0151_276	0.03475	low	Top Scoring Binders
16	0	0	0.0725	0.0625	0.303	0.0685	0.1185	0.107	0.058	0.048	0.0525	0.0615	-0.0005					11	89		0.45	277		2	3	Rv0146	Mb0151	topsharedbinders	5		#196	20	DAETAAIVAYLRSYQAVRTN	0	69	Mb0151_277	0.040375	low	Top Scoring Binders
17	0	0	0.065	0.0585	0.132	0.1015	0.0785	0.107	0.057	0.0445	0.05	0.0535	-0.0155					38	79		0.25	245		0	0	Rv0164	Mb0169	topsharedbinders	0		#36	20	DGRPSQVRLDTAVQGIEGTY	0	59	Mb0169_245	0.033125	low	Top Scoring Binders
261	0	0	0.063	0.0545	0.072	0.0705	0.167	0.105	0.057	0.0545	0.081	0.0855	-0.004					21	217		0.4	199		0	0	Rv3676	Mb3700	topsharedbinders	0		#252	20	FAHRGWIRLEGKSVLISDSE	0	197	Mb3700_199	0.05425	low	Top Scoring Binders
20	0	0	0.184	0.0515	0.166	0.069	0.0815	0.074	0.057	0.058	0.052	0.0595	-0.0015					9	245		0.5	252		1	2	Rv0241c	Mb0247c	topsharedbinders	3		#118	20	ARFPDAVRYSVRFAKPVLLP	0	225	Mb0247c_252	0.042	low	Top Scoring Binders
21	0	0	0.095	0.0605	0.08	0.0685	0.0845	0.077	0.07	0.0505	0.05	0.0575	0.0075					9	186		0.5	253		1	0	Rv0241c	Mb0247c	topsharedbinders	2		#136	20	LPPPAAVLRITPAKIRRYAA	0	166	Mb0247c_253	0.041375	low	Top Scoring Binders
259	0	0	0.14	0.052	0.229	0.0685	0.094	0.08	0.061	0.058	0.0505	0.056	-0.0015	5.477	0.2	2	12	5	127		0.45	40	100	1	1	Rv2054	Mb2080	cl_tepitopepan	2		#139	20	FCMGGQFALVLSPRGFGATA	0	115	Mb3695c_40	0.04075	low	Binder Clusters
45	0	0	0.0735	0.05	0.061	0.074	0.1875	0.077	0.058	0.051	0.0575	0.057	-0.0035					9	142		0.4	287		3	0	Rv0707	Mb0727	topsharedbinders	3		#206	20	LSNRVAFRRAMRKAIQSAMR	0	122	Mb0727_287	0.0405	low	Top Scoring Binders
46	0	0	0.1495	0.053	0.053	0.072	0.105	0.134	0.058	0.0515	0.085	0.074	0.0035					10	90		0.35	281		1	1	Rv0718	Mb0739	topsharedbinders	2		#134	20	ERSIAGLRRVSKPGLRVYAK	0	70	Mb0739_281	0.0535	low	Top Scoring Binders
187	0	0	0.1165	0.055	0.161	0.0735	0.1545	0.135	0.061	0.0775	0.0715	0.066	0.015	3.464	0.35	2	17	2	172	rmlB	0.45	70	100	0	1	Rv3464	Mb3493	cl_tepitopepan	1		#142	20	ADMLVRAWVRSYGVRATISN	0	155	Mb2963_70	0.0575	low	Binder Clusters
48	0	0	0.07	0.055	0.205	0.0855	0.12	0.093	0.056	0.046	0.0525	0.0605	-0.0025					8	46		0.4	228		1	0	Rv0720	Mb0741	topsharedbinders	1		#219	20	TAERPRLVVHRSARHIHVQL	0	26	Mb0741_228	0.039125	low	Top Scoring Binders
244	0	0	0.058	0.051	0.066	0.072	0.1345	0.075	0.057	0.0465	0.0545	0.124	-0.003	3.464	0.2	2	17	1	43		0.55	75	100	3	2	Rv2532c	Mb2561c	cl_tepitopepan	5		#203	20	CAAYGLTIVASAMSIYALGV	0	26	Mb3493_75	0.0555	low	Binder Clusters
243	0	3	0.082	0.0595	0.222	0.078	0.1265	0.189	0.062	0.2495	0.6005	0.56	0.006	6.481	0.25	3	15	7	79		0.5	115	100	1	0	Rv3463	Mb3492	cl_tepitopepan	1		#277	20	TTLCVATGIVNIWSAPAQRV	27	64	Mb3492_115	0.354	high	Binder Clusters
86	0	3	0.177	0.0585	0.319	0.075	0.0975	0.209	0.062	0.232	0.506	0.5755	0.0135	3.464	0.3	2	13	3	99	leuD	0.45	150	100	0	1	Rv2987c	Mb3011c	cl_netmhciipan	1		#173	20	ALMDYGFRVVISSRFGDIFR	27	86	Mb1538_150	0.33175	high	Binder Clusters
68	0	0	0.105	0.077	0.186	0.066	0.0705	0.078	0.055	0.084	0.051	0.055	-0.003	4	0.33	2	12	2	393	fadE19	0.39	64	100	0	2	Rv2500c	Mb2528c	cl_tepitopepan	2		#110	18	EGTTEVQLMLIARSLGLQ	0	381	Mb1402c_64	0.04675	low	Binder Clusters
69	0	0	0.105	0.0525	0.156	0.069	0.073	0.072	0.054	0.082	0.049	0.065	0.003	12.329	0.25	2	12	19	59		0.45	97	100	1	2	Rv1404	Mb1439	cl_netmhciipan	3		#164	20	ITIPQFRTLVILSNHGPINL	0	47	Mb1439_97	0.04975	low	Binder Clusters

47	0	1	0.183	0.0535	0.085	0.071	0.0845	0.083	0.059	0.2195	0.05	0.0565	-0.001					8	27		0.2	227		1	0	Rv0720	Mb0741	topsharedbinders	1	#135	20	TRRISRLRRHTRLRKKLSGT	9	7	Mb0741_227	0.08125	low	Top Scoring Binders
71	0	0	0.0515	0.0505	0.054	0.0885	0.102	0.067	0.058	0.1	0.064	0.075	-0.0095					41	63		0.45	247		0	0	Rv1436	Mb1471	topsharedbinders	0	#80	20	STLAHLLKFDSILGRLPCDV	0	43	Mb1471_247	0.057375	low	Top Scoring Binders
72	0	0	0.0765	0.0545	0.223	0.0725	0.0905	0.112	0.061	0.0465	0.104	0.0745	-0.0055					41	214		0.4	248		0	0	Rv1436	Mb1471	topsharedbinders	0	#76	20	DGPHKDLRRARAAALNIVPT	0	194	Mb1471_248	0.054875	low	Top Scoring Binders
73	0	0	0.072	0.103	0.222	0.083	0.1175	0.084	0.058	0.06	0.0505	0.053	-0.011					41	30		0.35	249		0	0	Rv1436	Mb1471	topsharedbinders	0	#23	20	GRIGRNFYRALLAQQEQGTA	0	10	Mb1471_249	0.038125	low	Top Scoring Binders
74	0	0	0.145	0.052	0.053	0.07	0.083	0.08	0.058	0.077	0.0495	0.0595	0.0025	12.806	0.3	2	16	41	80	gap	0.45	96	100	1	0	Rv1436	Mb1471	cl_netmhciipan	1	#113	20	DVGLEGDDTIVVGRAKIKAL	0	64	Mb1471_96	0.047125	low	Binder Clusters
83	0	1	0.0515	0.0515	0.092	0.0725	0.093	0.073	0.058	0.0465	0.186	0.1295	0.0055					89	171		0.4	231		0	0	Rv1488	Mb1524	topsharedbinders	0	#224	20	ATGRWGLRVARVELRSIDPP	9	151	Mb1524_231	0.091875	low	Top Scoring Binders
75	0	0	0.0715	0.0515	0.301	0.071	0.142	0.07	0.057	0.048	0.0515	0.0575	-0.0125	2.828	0.25	2	18	1	51	pks11	0.35	90	100	2	1	Rv1665	Mb1693	cl_tepitopepan	3	#56	20	KEHEEIIRRLHAAAKVNGRH	0	33	Mb1480c_90	0.036125	low	Binder Clusters
238	0	0	0.114	0.0565	0.123	0.072	0.09	0.077	0.059	0.0795	0.05	0.0635	0.001					14	66		0.5	212		0	0	Rv3443c	Mb3473c	topsharedbinders	0	#162	20	NVDGGDFVIVINADKVAISG	0	46	Mb3473c_212	0.0485	low	Top Scoring Binders
81	0	0	0.067	0.053	0.053	0.072	0.0745	0.074	0.058	0.0545	0.05	0.06	-0.001					23	216		0.25	262		0	0	Rv1479	Mb1515	topsharedbinders	0	#96	20	QRDRFLFKINVGYPSPEEER	0	196	Mb1515_262	0.040875	low	Top Scoring Binders
239	0	0	0.174	0.0635	0.263	0.068	0.1375	0.081	0.064	0.0785	0.049	0.056	0.009					14	82		0.2	213		0	0	Rv3443c	Mb3473c	topsharedbinders	0	#153	20	AISGDKLQHKMVYRHSGYPG	0	62	Mb3473c_213	0.048125	low	Top Scoring Binders
78	0	0	0.0635	0.0515	0.054	0.0715	0.0695	0.074	0.059	0.056	0.0495	0.0555	0.0055	9	0.3	3	17	3	157		0.45	12	100	4	2	Rv2715	Mb2734	cl_tepitopepan	6	#122	20	VTKDVNIVFRLASLPMGSEA	0	140	Mb1506_12	0.041625	low	Binder Clusters
247	0	0	0.0705	0.0505	0.241	0.0705	0.107	0.08	0.057	0.048	0.137	0.079	-0.012	3.162	0.3	2	10	1	72		0.35	154	100	1	1	Rv2286c	Mb2307c	cl_netmhciipan	2	#55	20	WSYGWSLMRIGALLRRTNMS	0	62	Mb3565c_154	0.063	low	Binder Clusters
245	0	0	0.086	0.053	0.101	0.078	0.172	0.084	0.062	0.0455	0.0545	0.054	-0.0095	6.481	0.15	2	18	7	243		0.5	116	100	1	1	Rv3520c	Mb3550c	cl_netmhciipan	2	#15	20	EDFEICATAQVVITDDRAAA	0	225	Mb3550c_116	0.036125	low	Binder Clusters
63	0	0	0.0675	0.0505	0.138	0.071	0.089	0.084	0.062	0.045	0.056	0.0665	-0.0005	4.243	0.2	2	16	3	185		0.45	59	100	1	1	Rv1260	Mb1290	cl_tepitopepan	2	#221	20	FGPEEQFVKRLGTHAAIFTV	0	169	Mb1290_59	0.04175	low	Binder Clusters
50	0	3	0.1755	0.0535	0.059	0.07	0.185	0.18	0.06	0.265	0.446	0.587	0.0365					10	195		0.45	203		0	1	Rv0757	Mb0780	topsharedbinders	1	#166	20	PTEFTLLRYFVINAGTVLSK	27	175	Mb0780_203	0.333625	high	Top Scoring Binders
51	0	2	0.063	0.052	0.06	0.0705	0.117	0.103	0.059	0.0735	0.199	0.17	-0.0115					10	93		0.35	204		0	0	Rv0757	Mb0780	topsharedbinders	0	#71	20	MPGMDGFGVLRRLRADGIDA	18	73	Mb0780_204	0.10775	low	Top Scoring Binders
52	0	0	0.0605	0.054	0.082	0.0735	0.1475	0.075	0.061	0.0475	0.0615	0.0615	-0.007					10	230		0.3	205		0	1	Rv0757	Mb0780	topsharedbinders	1	#69	20	VNVVESYVSYLRRKIDTGEK	0	210	Mb0780_205	0.040875	low	Top Scoring Binders
53	0	0	0.0995	0.054	0.112	0.0725	0.084	0.084	0.057	0.048	0.048	0.0555	0.0005					11	132		0.45	250		1	0	Rv0854	Mb0877	topsharedbinders	1	#129	20	QVPLPGFVLKRAIKGTIDTA	0	112	Mb0877_250	0.038	low	Top Scoring Binders
54	0	2	0.06	0.0865	0.051	0.0685	0.093	0.074	0.054	0.2715	0.252	0.139	0.0165					19	93		0.45	283		1	1	Rv1017c	Mb1045c	topsharedbinders	2	#172	20	WLMEQLIMIDALKRGSAKRI	18	73	Mb1045c_283	0.16975	low	Top Scoring Binders
55	0	0	0.084	0.0535	0.181	0.071	0.111	0.126	0.059	0.0465	0.06	0.061	0					19	292		0.3	284		0	0	Rv1017c	Mb1045c	topsharedbinders	0	#220	20	SCGAREVIVTNTLPIGEDKR	0	272	Mb1045c_284	0.041875	low	Top Scoring Binders
87	0	0	0.111	0.054	0.101	0.0675	0.1215	0.093	0.057	0.131	0.052	0.076	0.008	6.325	0.35	2	13	5	154	folD	0.4	32	99.6	1	0	Rv3356c	Mb3391c	cl_tepitopepan	1	#163	20	LPCTPRGIVHLLRRYDISIA	0	141	Mb1558_32	0.06675	low	Binder Clusters
56	0	0	0.1645	0.0535	0.205	0.0675	0.1225	0.106	0.063	0.082	0.0575	0.064	0.0085	5.292	0.3	2	16	2	116	sugB	0.55	127	100	2	4	Rv1237	Mb1269	cl_netmhciipan	6	#154	20	GKRLLIGAALLITMFPSISL	0	100	Mb1269_127	0.053	low	Binder Clusters
58	0	0	0.0785	0.055	0.091	0.0695	0.1105	0.09	0.063	0.044	0.0495	0.052	-0.009	4.899	0.25	2	16	6	75		0.35	52	100	1	0	Rv2454c	Mb2481c	cl_tepitopepan	1	#44	20	PGCGDYVILNTIRNFLPELG	0	59	Mb1270_52	0.034125	low	Binder Clusters
249	0	0	0.071	0.0745	0.228	0.084	0.1335	0.105	0.06	0.062	0.0605	0.054	-0.006	3.464	0.25	2	12	2	18	add	0.45	79	100	2	0	Rv3313c	Mb3342c	cl_tepitopepan	2	#24	20	TAAPTLQTIRLAPKALLHDH	0	6	Mb3606c_79	0.042625	low	Binder Clusters
60	0	1	0.059	0.05	0.072	0.0685	0.1645	0.092	0.058	0.0465	0.1365	0.166	-0.0015	4.243	0.35	2	12	3	248		0.45	137	100	0	1	Rv1421	Mb1456	cl_netmhciipan	1	#225	20	GAAEFLESYHRLLSLVVDGY	9	236	Mb1271c_137	0.086875	low	Binder Clusters
248	0	0	0.086	0.051	0.09	0.0745	0.115	0.09	0.057	0.046	0.061	0.0585	-0.004	2.449	0.35	3	20	1	225	parA	0.55	173	100	0	1	Rv3918c	Mb3949c	cl_netmhciipan	1	#259	20	YVFVDCPPSLGLLTINALVA	0	205	Mb3576_173	0.040375	low	Binder Clusters
62	0	0	0.0705	0.051	0.31	0.0705	0.134	0.086	0.06	0.0465	0.059	0.0665	-0.012	3.464	0.25	2	18	1	146		0.4	149	100	1	3	Rv1500	Mb1538	cl_netmhciipan	4	#54	20	GWLRNFGPKIHYRASALLCD	0	128	Mb1277c_149	0.04	low	Binder Clusters
250	0	3	0.2245	0.0565	0.174	0.074	0.2015	0.23	0.06	0.25	0.301	0.656	0.025					7	69		0.45	263		0	0	Rv3584	Mb3615	topsharedbinders	0	#174	20	LTINNVLLRDIRIQAVQTSD	27	49	Mb3615_263	0.308	high	Top Scoring Binders
79	0	0	0.058	0.0525	0.195	0.071	0.207	0.074	0.06	0.0515	0.1005	0.15	-0.0085					23	353		0.45	259		2	0	Rv1479	Mb1515	topsharedbinders	2	#251	20	DEISPEIVINRVLQTVALPQ	0	333	Mb1515_259	0.073375	low	Top Scoring Binders
237	1	0	0.2195	0.0545	0.054	0.0855	0.4385	0.131	0.061	0.095	0.049	0.056	0.013					14	131		0.25	211		0	0	Rv3443c	Mb3473c	topsharedbinders	0	#128	20	NRLSRQIQRKLRVYAGPEHP	9	111	Mb3473c_211	0.05325	low	Top Scoring Binders
274	1	0	0.0965	0.0545	0.065	0.0835	0.2655	0.093	0.061	0.051	0.079	0.066	0.0075	9.381	0.35	2	12	11	22		0.45	8	100	1	1	Rv2949c	Mb2973c	cl_tepitopepan	2	#177	20	LSDQEIRKLNRDLRILIAAN	9	10	Mb3876_8	0.050875	low	Binder Clusters
236	1	0	0.0805	0.136	0.425	0.084	0.2225	0.133	0.055	0.0485	0.0615	0.0685	-0.0055					14	119		0.4	210		0	0	Rv3443c	Mb3473c	topsharedbinders	0	#239	20	VVEKAILGMLPKNRLSRQIQ	9	99	Mb3473c_210	0.04325	low	Top Scoring Binders
265	1	0	0.0925	0.055	0.216	0.0755	0.2665	0.096	0.064	0.0665	0.048	0.0565	0.0035	6.481	0.35	2	19	7	155	moxR2	0.45	117	100	1	0	Rv3692	Mb3717	cl_netmhciipan	1	#137	20	VFRPGPVFTNLLLADEINRT	9	136	Mb3717_117	0.043625	low	Binder Clusters
234	1	2	0.061	0.097	0.293	0.1965	0.1335	0.24	0.07	0.0645	0.255	0.379	-0.011	6.928	0.25	2	17	6	117		0.4	23	100	2	1	Rv1463	Mb1498	cl_tepitopepan	3	#73	20	VPGVSMSNFLRSAATAIRGE	27	100	Mb3422_23	0.171875	high	Binder Clusters
242	1	1	0.081	0.1155	0.608	0.172	0.115	0.122	0.062	0.048	0.118	0.3215	-0.009					60	89		0.4	209		0	0	Rv3457c	Mb3486c	topsharedbinders	0	#58	20	KEDVTEIILNLKSLVVSSEE	18	69	Mb3486c_209	0.119625	low	Top Scoring Binders
241	1	0	0.0655	0.088	0.481	0.1645	0.096	0.126	0.06	0.0465	0.06	0.11	-0.009					60	67		0.45	208		0	1	Rv3457c	Mb3486c	topsharedbinders	1	#60	20	GAAVTSIRIDGVLHEFTTVP	9	47	Mb3486c_208	0.051875	low	Top Scoring Binders
229	1	0	0.0655	0.065	0.186	0.0705	0.535	0.087	0.061	0.057	0.081	0.062	-0.0015	4	0.3	2	12	2	157	add	0.35	142	100	1	2	Rv3313c	Mb3342c	cl_netmhciipan	3	#198	20	GQPITVRCLVTAMRHAAMSR	9	145	Mb3329c_142	0.049625	low	Binder Clusters
199	1	0	0.169	0.0585	0.166	0.072	0.507	0.1	0.061	0.066	0.06	0.076	0.0075					8	88		0.55	272		1	0	Rv3000	Mb3025	topsharedbinders	1	#141	20	LTDLDVLVLRRRVGLVAQAP	9	68	Mb3025_272	0.052375	low	Top Scoring Binders
262	1	4	0.209	0.0575	0.053	0.072	0.118	0.519	0.072	0.9865	1.587	1.426	0.182					21	137		0.35	200		0	1	Rv3676	Mb3700	topsharedbinders	1	#257	20	EISEQLLRVLARRLRRTNNN	45	117	Mb3700_200	1.045375	high	Top Scoring Binders
224	1	0	0.068	0.054	0.124	0.071	0.6285	0.109	0.061	0.0475	0.1305	0.091	0.0005	3.464	0.3	3	18	1	248	drrB	0.55	147	100	2	0	Rv2937	Mb2962	cl_netmhciipan	2	#204	20	HPFVRNQPISQFVAALRALA	9	230	Mb3275c_147	0.067375	low	Binder Clusters
206	1	0	0.0775	0.144	0.153	0.3455	0.122	0.18	0.064	0.057	0.0555	0.0795	-0.0135	4	0.25	2	14	4	106	phoY1	0.35	141	100	1	0	Rv3301c	Mb3329c	cl_netmhciipan	1	#33	20	DTERMGALAVHIAKITRREY	9	92	Mb3131c_141	0.044625	low	Binder Clusters
207	1	2	0.07	0.0565	0.065	0.067	0.554	0.187	0.065	0.0585	0.352	0.237	-0.0045	5.477	0.35	2	12	5	304	prfB	0.45	125	100	1	1	Rv3105c	Mb3132c	cl_netmhciipan	2	#191	20	QLQNKIAAMRVLQAKLLERK	27	292	Mb3132c_125	0.16075	high	Binder Clusters
209	1	0	0.076	0.0575	0.11	0.085	0.251	0.074	0.062	0.0455	0.055	0.0545	-0.0105					6	37		0.25	275		0	1	Rv3143	Mb3167	topsharedbinders	1	#41	20	QTRERVMRALGKRLHPDLPD	9	17	Mb3167_275	0.036125	low	Top Scoring Binders
210	1	3	0.0885	0.0535	0.059	0.07	0.3685	0.11	0.059	0.3185	0.6555	0.3745	0.006	3.162	0.25	2	16	1	151	nuoB	0.35	83	100	1	1	Rv3146	Mb3170	cl_tepitopepan	2	#178	20	PPRPEMLLHAILKLHEKIQQ	36	135	Mb3170_83	0.338625	high	Binder Clusters
251	1	0	0.0615	0.089	0.497	0.0685	0.0765	0.087	0.055	0.0465	0.053	0.066	-0.0055					7	92		0.45	264		0	0	Rv3584	Mb3615	topsharedbinders	0	#211	20	PGKAVDLVLVAVNQSPDVSD	9	72	Mb3615_264	0.04	low	Top Scoring Binders
240	1	0	0.0945	0.056	0.249	0.0735	0.5455	0.119	0.06	0.0565	0.1465	0.0795	0.0085	5.477	0.3	2	14	3	306	sugC	0.55	41	100	2	1	Rv1238	Mb1270	cl_tepitopepan	3	#175	20	DAALIDAYQRIRALTFQVKV	9	292	Mb3484c_41	0.07275	low	Binder Clusters
252	1	3	0.077	0.0515	0.155	0.0715	0.088	0.26	0.062	0.525	0.878	0.818	0.0705					7	63		0.45	265		0	0	Rv3584	Mb3615	topsharedbinders	0	#269	20	AVNGNRLTINNVLLRDIRIQ	36	43	Mb3615_265	0.572875	high	Top Scoring Binders
0	1	2	0.0755	0.0545	0.347	0.0855	0.1225	0.124	0.063	0.07	0.3815	0.521	0.004									300						_pos ctrls					27		CFP10.1_300	0.244125	high	_pos ctrls
179	1	1	0.106	0.055	0.217	0.072	0.2435	0.133	0.055	0.0525	0.316	0.1295	-0.002					23	155		0.35	194		0	0	Rv2803	Mb2826	topsharedbinders	0	#240	20	DEAAPELVRLARRKSRIPPH	18	135	Mb2826_194	0.124	low	Top Scoring Binders
144	1	0	0.2875	0.0585	0.269	0.0755	0.1295	0.13	0.06	0.0625	0.0495	0.0695	0.0055	2.449	0.35	2	14	1	134	aroB	0.5	178	100	1	1	Rv2538c	Mb2567c	cl_netmhciipan	2	#161	20	AATWLRGVSIVHLPTTLLGM	9	120	Mb2307c_178	0.04675	low	Binder Clusters
57	1	0	0.0585	0.0585	0.179	0.0875	0.2405	0.093	0.071	0.057	0.1505	0.123	-0.0055	5.292	0.25	4	20	1	319	murX	0.3	45	100	3	2	Rv2156c	Mb2180c	cl_tepitopepan	5	#89	20	ILTFRTTGRRMFRMAPFHHH	9	299	Mb1270_45	0.08125	low	Binder Clusters
64	1	1	0.082	0.0575	0.345	0.1055	0.18	0.096	0.063	0.0505	0.107	0.483	-0.005	3.162	0.35	2	18	1	240		0.45	82	99.7	0	1	Rv2426c	Mb2450c	cl_tepitopepan	1	#84	20	HFAEELVRIIGVLRGMQLKK	18	222	Mb1298c_82	0.158875	low	Binder Clusters
137	1	0	0.1585	0.0615	0.13	0.073	0.509	0.135	0.061	0.0785	0.1385	0.081	0.034	2.828	0.35	2	12	1	133		0.5	162	100	1	1	Rv2212	Mb2235	cl_netmhciipan	2	#176	20	GEDGAFGLLRVLGTAMARLA	9	121	Mb2235_162	0.083	low	Binder Clusters
136	1	0	0.081	0.071	0.205	0.07	0.319	0.107	0.061	0.0465	0.0525	0.059	0.0025	7.746	0.33	3	21	5	146	lipV	0.43	19		2	1	Rv3203		cl_tepitopepan	3	#222	21	EHLVALPNGRYGWRISLPAMV	9	125	Mb2233c_19	0.040125	low	Binder Clusters
65	1	0	0.0765	0.081	1.312	0.0725	0.176	0.097	0.061	0.0595	0.0535	0.052	-0.0105	2	0.15	2	17	1	204		0.3	187	100	0	1	Rv2165c	Mb2189c	cl_netmhciipan	1	#31	20	FAYATDAPLDMRMDPTTPLT	9	187	Mb1320_187	0.038625	low	Binder Clusters
128	1	2	0.096	0.0585	0.168	0.074	0.344	0.196	0.062	0.1775	0.288	0.0625	0.0165	3.162	0.25	2	11	1	242		0.4	155	100	2	2	Rv2516c	Mb2545c	cl_netmhciipan	4	#276	20	RPGEGLNMVLIAAWGHPLPN	27	231	Mb2180c_155	0.136125	high	Binder Clusters
93	1	2	0.0695	0.0725	0.371	0.12	0.1795	0.095	0.061	0.0895	0.4845	0.34	0.006	10.583	0.25	2	15	7	375		0.45	5	99.7	2	2	Rv3161c	Mb3186c	cl_tepitopepan	4	#90	20	KEWPGMLAFYRNLAMALVGD	27	360	Mb1620_5	0.23	high	Binder Clusters
66	1	0	0.073	0.0515	0.076	0.0745	0.2595	0.101	0.057	0.051	0.057	0.064	-0.002	9.165	0.15	2	17	7	84	trxB1	0.5	11	100	2	3	Rv1471	Mb1506	cl_tepitopepan	5	#202	20	AAQIRSIPTIMAFKNGKLLF	9	67	Mb1328_11	0.0425	low	Binder Clusters
118	1	2	0.408	0.0545	0.068	0.0705	0.13	0.115	0.067	0.2575	0.1055	0.289	0.039					49	285		0.4	230		1	2	Rv1886c	Mb1918c	topsharedbinders	3	#179	20	AEFLENFVRSSNLKFQDAYN	27	265	Mb1918c_230	0.17275	high	Top Scoring Binders
76	1	0	0.0585	0.0735	0.524	0.1025	0.139	0.169	0.059	0.0475	0.0815	0.096	-0.011	4	0.25	2	12	4	82	argB	0.3	62	100	0	1	Rv1654	Mb1682	cl_tepitopepan	1	#66	20	VHGGGPQITAMLRRLGIEGD	9	70	Mb1481c_62	0.0535	low	Binder Clusters
112	1	0	0.08	0.057	0.078	0.068	0.3625	0.103	0.055	0.0455	0.0605	0.059	-0.0055	2	0.25	2	10	1	65	era	0.3	188	100	1	0	Rv2364c	Mb2385c	cl_netmhciipan	1	#246	20	HSDDFQIILVDTPGLHRPRT	9	55	Mb1870c_188	0.039875	low	Binder Clusters
82	1	2	0.0635	0.0515	0.277	0.071	0.1365	0.291	0.062	0.119	0.32	0.1995	-0.007	10.583	0.25	2	16	7	314	nadA	0.35	4	100	4	1	Rv1594	Mb1620	cl_tepitopepan	5	#68	20	DRASCKYMKMITPAALLRCL	27	298	Mb1520_4	0.157875	high	Binder Clusters
85	1	3	0.1805	0.054	0.129	0.08	0.1515	0.438	0.066	0.4055	0.9395	0.9785	0.0915					23	92		0.55	229		0	1	Rv1489	Mb1525	topsharedbinders	1	#265	20	TAMLTLYFILAVGAHVRVRD	36	72	Mb1525_229	0.60375	high	Top Scoring Binders
181	1	0	0.07	0.1415	0.133	0.214	0.1545	0.153	0.064	0.0495	0.095	0.134	-0.0135	2.828	0.35	2	16	2	304	mutT1	0.4	94	100	0	1	Rv2985	Mb3009	cl_tepitopepan	1	#34	20	NRKGSTWVLSLSAGRLVTAD	9	288	Mb2842c_94	0.06625	low	Binder Clusters
91	1	0	0.0775	0.0525	0.358	0.0705	0.138	0.094	0.056	0.046	0.0525	0.0585	-0.002					51	49		0.4	236		0	1	Rv1544	Mb1571	topsharedbinders	1	#212	20	AGRGFPLMLVARRRERLDEL	9	29	Mb1571_236	0.03875	low	Top Scoring Binders
120	1	3	0.0955	0.0755	0.086	0.0735	0.1065	0.295	0.063	0.597	1.0985	1.361	0.0875	6.481	0.25	2	12	7	42	asd	0.45	28	100	1	1	Rv3708c	Mb3735c	cl_tepitopepan	2	#256	20	DFPASAVRFFASARSQGRKL	36	30	Mb2000_28	0.786	high	Binder Clusters
155	1	4	0.139	0.1065	0.082	0.0725	0.1025	0.462	0.069	0.595	0.716	0.932	0.1015	6.325	0.2	2	15	10	86	ahpD	0.45	118	100	0	1	Rv2429	Mb2455	cl_netmhciipan	1	#215	20	HAALGAAAIMGMNNVFYRGR	45	71	Mb2455_118	0.586125	high	Binder Clusters
49	1	0	0.066	0.0555	0.201	0.079	0.3455	0.092	0.058	0.0485	0.0535	0.0575	-0.01					10	145		0.4	202		0	1	Rv0757	Mb0780	topsharedbinders	1	#61	20	EEVVARLRVILRRAGKGNKE	9	125	Mb0780_202	0.037375	low	Top Scoring Binders
158	1	0	0.0965	0.0695	0.358	0.0665	0.1035	0.095	0.059	0.0635	0.072	0.103	0.002	6.928	0.35	3	18	4	136		0.4	24	100	1	2	Rv3921c	Mb3952c	cl_tepitopepan	3	#165	20	PVFLGLYHVLRSFNRTTGGF	9	118	Mb2481c_24	0.060125	low	Binder Clusters
1	1	2	0.095	0.052	0.243	0.0715	0.2965	0.106	0.07	0.058	0.179	1.5785	-0.003									301						_pos ctrls					27		CFP10.2_301	0.453125	high	_pos ctrls
176	1	0	0.175	0.0515	0.079	0.0675	0.2795	0.086	0.062	0.074	0.0615	0.0625	0.018	3.873	0.35	3	20	1	46	drrB	0.4	68	100	3	1	Rv2937	Mb2962	cl_tepitopepan	4	#140	20	STGRQMWVLYRRFAAPSLLN	9	26	Mb2772_68	0.054	low	Binder Clusters
19	1	0	0.0635	0.0525	0.41	0.067	0.13	0.094	0.062	0.047	0.0575	0.062	-0.0065					9	136		0.35	251		1	0	Rv0241c	Mb0247c	topsharedbinders	1	#213	20	REHRRGLLVDLVTNVSVGND	9	116	Mb0247c_251	0.04	low	Top Scoring Binders
174	1	2	0.082	0.107	0.142	0.0835	0.084	0.411	0.064	0.0935	0.584	0.441	0.031	2.828	0.3	2	18	1	214		0.35	167	99.4	1	2	Rv2714	Mb2733	cl_netmhciipan	3	#236	20	LEYRMAQHGHEVVGFTVHVP	27	196	Mb2733_167	0.287375	high	Binder Clusters
173	1	0	0.1185	0.053	0.05	0.066	0.335	0.077	0.061	0.0475	0.05	0.0585	0.0005	4	0.3	2	18	2	148		0.5	140	100	2	1	Rv3104c	Mb3131c	cl_netmhciipan	3	#155	20	QRLVQDLLAGFFIIVEKQYG	9	130	Mb2649_140	0.039125	low	Binder Clusters
172	1	0	0.0735	0.0505	0.366	0.0695	0.155	0.083	0.058	0.1035	0.055	0.0555	-0.011	2.449	0.35	2	12	1	69	sdhB	0.4	180	99.6	1	0	Rv3319	Mb3348	cl_netmhciipan	1	#72	20	LPSDRLLNLLIYIKGYLDGT	9	57	Mb2645c_180	0.05075	low	Binder Clusters
171	1	4	0.2265	0.0585	0.15	0.0775	0.1115	0.718	0.107	0.5845	1.2925	1.0605	0.114	5.657	0.3	2	16	4	199	tesB2	0.45	122	99.6	1	2	Rv2605c	Mb2637c	cl_netmhciipan	3	#235	20	DPVLHICALAYMSDLTLLGS	45	183	Mb2637c_122	0.762875	high	Binder Clusters
43	1	0	0.0715	0.116	0.467	0.0805	0.222	0.09	0.058	0.047	0.0945	0.0985	-0.01					9	76		0.35	285		0	0	Rv0707	Mb0727	topsharedbinders	0	#79	20	ERTRDRVRVDIHTARPGIVI	9	56	Mb0727_285	0.0575	low	Top Scoring Binders
97	1	0	0.0815	0.0515	0.32	0.071	0.2595	0.103	0.06	0.048	0.0575	0.066	-0.004					10	187		0.3	216		2	2	Rv1626	Mb1652	topsharedbinders	4	#199	20	MTEPDAFKWIQRAAMDRRTT	9	167	Mb1652_216	0.041875	low	Top Scoring Binders
169	1	3	0.1055	0.063	0.099	0.0695	0.1385	0.277	0.07	0.201	0.616	0.464	0.018					7	57		0.25	269		2	2	Rv2579	Mb2610	topsharedbinders	4	#274	20	GNPTSSYLWRNIMPHLEGLG	36	37	Mb2610_269	0.32475	high	Top Scoring Binders
30	1	0	0.072	0.0605	0.352	0.072	0.149	0.076	0.06	0.0945	0.067	0.089	-0.0105					8	80		0.6	280		0	0	Rv0500	Mb0511	topsharedbinders	0	#78	20	AVENATFVVVAVKPADVEPV	9	60	Mb0511_280	0.06	low	Top Scoring Binders
36	1	4	0.064	0.051	0.268	0.066	1.0515	0.21	0.059	0.6535	1.8655	2.324	0.1085					39	251		0.35	220		1	2	Rv0655	Mb0674	topsharedbinders	3	#192	20	DNMGMLFRKHLVMFGPREVL	45	231	Mb0674_220	1.237875	high	Top Scoring Binders
165	1	1	0.142	0.0615	0.164	0.075	0.2495	0.125	0.059	0.154	0.137	0.131	0.0195	2.828	0.3	2	11	1	181	mrr	0.4	166	99.7	1	1	Rv2528c	Mb2557c	cl_netmhciipan	2	#97	20	TGFEDLVIRLLEAMGYGRAG	18	170	Mb2557c_166	0.110375	low	Binder Clusters
37	1	0	0.0935	0.0625	0.356	0.0755	0.125	0.104	0.072	0.045	0.0895	0.069	-0.0005					39	86		0.5	221		0	0	Rv0655	Mb0674	topsharedbinders	0	#8	20	SVFLKSLIGLLRPERGSIII	9	66	Mb0674_221	0.05075	low	Top Scoring Binders
159	1	0	0.2985	0.0525	0.237	0.08	0.1105	0.075	0.058	0.112	0.0505	0.0685	-0.0005	4.899	0.3	2	19	3	128	cdh	0.5	50	100	2	1	Rv2289	Mb2311	cl_tepitopepan	3	#123	20	NYLYDAWAARFLVTARLNNS	9	109	Mb2481c_50	0.057625	low	Binder Clusters
246	2	4	0.1095	0.051	0.329	0.086	0.8625	0.442	0.067	0.4675	0.8615	0.387	0.1105	9.95	0.25	3	15	11	26		0.4	104	100	1	1	Rv3528c	Mb3558c	cl_netmhciipan	2	#193	20	GGYWLVRGKINLIDRAFTSC	55	11	Mb3558c_104	0.456625	high	Binder Clusters
80	2	4	0.117	0.0565	0.547	0.0665	0.2395	0.252	0.065	0.415	1.354	0.99	0.1175					23	334		0.5	261		0	0	Rv1479	Mb1515	topsharedbinders	0	#200	20	EVIPDVLRHRLVLTYDALAD	55	314	Mb1515_261	0.719125	high	Top Scoring Binders
38	2	0	0.081	0.175	0.372	0.23	0.135	0.138	0.06	0.049	0.0545	0.071	-0.008					39	271		0.35	222		0	0	Rv0655	Mb0674	topsharedbinders	0	#50	20	LTSDEPVVRQFLNGRRIGPI	18	251	Mb0674_222	0.041625	low	Top Scoring Binders
18	2	0	0.0755	0.078	0.346	0.0735	0.318	0.085	0.069	0.0535	0.056	0.0585	-0.0045					38	148		0.4	246		0	0	Rv0164	Mb0169	topsharedbinders	0	#32	20	MPVPEPMVKMLLNNVLEHLA	18	128	Mb0169_246	0.040875	low	Top Scoring Binders
22	2	0	0.0565	0.054	0.742	0.0675	0.4715	0.093	0.065	0.048	0.0865	0.0785	-0.0065					18	44		0.4	239		0	0	Rv0247c	Mb0253c	topsharedbinders	0	#197	20	EVNEGEVVLDVILRLQQTQT	18	24	Mb0253c_239	0.051625	low	Top Scoring Binders
70	2	2	0.1795	0.0555	0.375	0.08	0.6915	0.207	0.068	0.096	0.1715	0.2325	0.0045	4.243	0.3	2	13	3	124	parB	0.3	138	100	1	0	Rv3917c	Mb3948c	cl_netmhciipan	1	#158	20	SQTGVRYQIVMGERRWRAAQ	36	111	Mb1456_138	0.126125	high	Binder Clusters
235	2	1	0.0695	0.055	0.418	0.0805	0.282	0.138	0.07	0.0465	0.106	0.1945	-0.004	12	0.25	4	20	6	198	lpqD	0.5	98	100	3	1	Rv3390	Mb3422	cl_netmhciipan	4	#210	20	VVFSQGVAIMIWTLMNARNS	27	178	Mb3422_98	0.08575	high	Binder Clusters
230	2	2	0.0765	0.052	0.058	0.07	0.338	0.26	0.065	0.0485	0.4055	0.247	-0.0545	4	0.35	2	10	4	68		0.35	143	100	0	1	Rv3921c	Mb3952c	cl_netmhciipan	1	#273	20	ALLYKPFVRQIRTTRQMQEL	36	58	Mb3342c_143	0.161625	high	Binder Clusters
190	2	2	0.1425	0.092	0.454	0.0885	0.2745	0.161	0.07	0.134	0.3705	0.9845	-0.002	6	0.3	2	16	9	148		0.2	34	99.7	0	1	Rv3200c	Mb3225c	cl_tepitopepan	1	#38	20	ETSRQAYKIQRWRSRVRNHT	36	132	Mb2978c_34	0.37175	high	Binder Clusters
191	2	0	0.1835	0.0825	0.548	0.11	0.2725	0.179	0.068	0.062	0.055	0.1125	0.005	4	0.3	2	16	2	188	opcA	0.5	60	99.7	1	2	Rv1446c	Mb1481c	cl_tepitopepan	3	#159	20	TDLAWSRITYWRALLTSAVD	18	172	Mb2978c_60	0.058625	low	Binder Clusters
103	2	3	0.138	0.0785	0.252	0.073	0.595	0.469	0.074	0.2955	0.7185	0.678	0.0435	7.071	0.2	2	10	5	120		0.45	107	100	2	2	Rv1676	Mb1703	cl_netmhciipan	4	#241	20	DSGITEVVFFHSAADALRGY	45	110	Mb1703_107	0.433875	high	Binder Clusters
162	2	4	0.2135	0.055	0.327	0.0715	0.385	0.482	0.058	0.236	2.294	1.1365	0.232	4.243	0.25	3	20	3	189		0.45	134	100	0	1	Rv2172c	Mb2194c	cl_netmhciipan	1	#232	20	YGMTQLLYSDAIVGFLREFA	55	169	Mb2539_134	0.974625	high	Binder Clusters
204	2	2	0.131	0.228	0.266	0.2705	0.084	0.166	0.069	0.0915	0.2655	0.4875	-0.0125	2.828	0.3	2	17	1	38	argR	0.4	89	100	1	2	Rv1657	Mb1685	cl_tepitopepan	3	#29	20	GRQARIVAILSSAQVRSQNE	36	21	Mb3098_89	0.208	high	Binder Clusters
157	2	1	0.07	0.056	0.284	0.0725	0.595	0.285	0.067	0.1215	0.316	0.0655	-0.015	7.746	0.35	2	20	6	111	ilvE	0.5	18	99.7	1	2	Rv2210c	Mb2233c	cl_tepitopepan	3	#272	20	SFRADANAARLRSSARRLAI	27	91	Mb2480c_18	0.122	high	Binder Clusters
152	2	0	0.083	0.056	0.367	0.1065	0.44	0.104	0.058	0.048	0.0755	0.0595	0	3.464	0.25	2	16	2	37	argC	0.4	73	100	1	0	Rv1652	Mb1680	cl_tepitopepan	1	#229	20	GYAGGEILRLLLGHPAYADG	18	21	Mb2435_73	0.04575	low	Binder Clusters
141	2	0	0.077	0.245	0.359	0.1325	0.1895	0.105	0.068	0.045	0.0685	0.0695	-0.006	2.449	0.35	2	12	1	199	lipA	0.35	177	100	0	1	Rv2218	Mb2241	cl_netmhciipan	1	#86	20	TVPRIFKRIRPAFTYRRSLG	18	187	Mb2241_177	0.04425	low	Binder Clusters
212	2	4	0.2525	0.057	0.14	0.0725	0.218	0.491	0.067	0.8025	1.3955	1.5315	0.174	5.657	0.15	2	15	2	60	nuoJ	0.55	123	100	4	3	Rv3154	Mb3178	cl_netmhciipan	7	#249	20	YSAMFLAMTMIILAVFYMAQ	55	45	Mb3178_123	0.975875	high	Binder Clusters
215	2	4	0.383	0.054	0.226	0.0735	0.1535	0.664	0.063	1.117	1.736	2.0845	0.271	4	0.35	2	19	2	223		0.5	63	100	0	2	Rv2417c	Mb2440c	cl_tepitopepan	2	#263	20	TAVMIDRVCQLVGDRPAALA	55	204	Mb3223_63	1.302125	high	Binder Clusters
281	2	3	0.107	0.054	0.626	0.1195	0.1545	0.336	0.082	0.2045	0.642	0.555	0.038	6.708	0.2	3	20	5	56	ftsZ	0.55	26	100	1	1	Rv2150c	Mb2174c	cl_tepitopepan	2	#67	20	FIAINTDAQALLMSDADVKL	45	36	Mb3952c_26	0.359875	high	Binder Clusters
105	2	2	0.1795	0.2785	0.161	0.3665	0.0945	0.212	0.086	0.1335	0.303	0.7735	-0.0055	3	0.35	3	20	1	321		0.5	158	100	1	0	Rv3071	Mb3098	cl_netmhciipan	1	#43	20	FQLVRRVPVTTIIVDTPQAI	36	301	Mb1735_158	0.301125	high	Binder Clusters
222	2	4	0.2675	0.0535	0.199	0.073	0.101	0.455	0.064	0.941	1.131	1.5465	0.219					17	175		0.5	223		0	1	Rv3246c	Mb3274c	topsharedbinders	1	#255	20	PLEFDLLVALARKPRQVFTR	55	155	Mb3274c_223	0.959375	high	Top Scoring Binders
270	3	0	0.306	0.108	0.368	0.081	0.4865	0.172	0.066	0.097	0.0525	0.0835	0.004	2	0.3	2	14	1	63	vapB13	0.35	185	100	1	0	Rv1839c	Mb1870c	cl_netmhciipan	1	#100	20	GDLDMKLRSVRAAARHEFPT	27	49	Mb3759_185	0.05925	high	Binder Clusters
11	3	4	0.201	0.1755	0.766	0.0695	0.739	0.463	0.111	0.716	2.4735	0.863	0.619									311						_pos ctrls					64		ESAT-6 p55_311	1.167875	high	_pos ctrls
260	3	3	0.1035	0.053	0.194	0.07	0.3125	0.281	0.306	0.4355	0.693	0.858	0.011					21	62		0.35	198		0	0	Rv3676	Mb3700	topsharedbinders	0	#82	20	PGDRLYIIISGKVKIGRRAP	55	42	Mb3700_198	0.499375	high	Top Scoring Binders
198	3	0	0.093	0.0935	1.881	0.263	0.741	0.118	0.06	0.0495	0.062	0.067	-0.0015					8	186		0.6	271		3	2	Rv3000	Mb3025	topsharedbinders	5	#223	20	VDPLTAIRYQIVVMYLLLAA	27	166	Mb3025_271	0.04425	high	Top Scoring Binders
26	3	0	0.2365	0.3525	0.739	0.182	0.5465	0.18	0.067	0.0985	0.069	0.074	0.0025					15	87		0.4	225		1	1	Rv0464c	Mb0473c	topsharedbinders	2	#116	20	PGVDTELVILRVAHLRSCEY	27	67	Mb0473c_225	0.061	high	Top Scoring Binders
3	3	2	0.091	1.08	0.256	0.2395	0.3535	0.145	0.069	0.0865	0.563	0.9135	0.0035									303						_pos ctrls					45		CFP10.8_303	0.391625	high	_pos ctrls
106	3	4	0.327	0.0545	0.054	0.0695	0.4285	0.657	0.077	0.7315	1.3355	1.2005	0.1335	2.828	0.2	2	18	2	129		0.4	161	100	1	0	Rv1762c	Mb1793c	cl_netmhciipan	1	#180	20	EHLEFMAVGTAVRYTAKPGA	64	111	Mb1793c_161	0.85025	high	Binder Clusters
254	3	3	0.066	0.0495	0.355	0.0775	0.515	0.3	0.067	0.449	0.956	0.445	0.0775	4.899	0.3	2	18	3	165	sugC	0.4	51	100	0	2	Rv1238	Mb1270	cl_tepitopepan	2	#268	20	GRAIVRHPKAFLMDEPLSNL	55	147	Mb3633c_51	0.481875	high	Binder Clusters
226	3	4	0.212	0.061	0.498	0.074	0.6285	0.528	0.08	0.776	2.6	1.9205	0.2535	5.657	0.35	2	12	8	166		0.4	37	100	0	1	Rv1833c	Mb1864c	cl_tepitopepan	1	#242	20	VMSSPPVQYAILRRNFFVER	64	154	Mb3300_37	1.3875	high	Binder Clusters
107	3	4	0.363	0.068	0.228	0.0825	0.2605	0.725	0.093	0.8205	2.561	2.078	0.66	2.828	0.3	2	13	1	171		0.5	85	100	1	0	Rv1591	Mb1617	cl_tepitopepan	1	#278	20	TQAPPVFFARRPLQIALTLM	64	158	Mb1853_85	1.529875	high	Binder Clusters
277	3	0	0.154	0.1395	1.278	0.292	0.6585	0.2	0.075	0.0515	0.079	0.091	0	2.449	0.25	3	15	1	21	apt	0.45	171	99.6	0	1	Rv2584c	Mb2615c	cl_netmhciipan	1	#157	20	GGTWAGDYVLNVIATGLSLK	27	6	Mb3947c_171	0.055375	high	Binder Clusters
77	3	3	0.2335	0.3145	0.148	0.3055	0.1775	0.285	0.101	0.2085	0.682	1.257	0.02	6.928	0.2	2	14	6	199	lpqD	0.35	25	100	2	0	Rv3390	Mb3422	cl_tepitopepan	2	#30	20	SQGVAIMIWTLMNARNSRDS	55	185	Mb1498_25	0.541875	high	Binder Clusters
61	3	3	0.1855	0.2365	0.132	0.075	0.3635	0.332	0.062	0.4	0.476	0.19	0.078					46	32		0.5	257		3	2	Rv1240	Mb1272	topsharedbinders	5	#156	20	AAGQIGYSLLFRLASGSLLG	55	12	Mb1272_257	0.286	high	Top Scoring Binders
131	3	0	0.175	0.1865	1.437	0.179	0.9005	0.378	0.084	0.069	0.1475	0.1375	-0.0105	4.243	0.2	3	15	2	138		0.3	135	99.4	1	1	Rv2256c	Mb2280c	cl_netmhciipan	2	#45	20	GDIMVGEMRLRAMMAQGADT	27	123	Mb2194c_135	0.085875	high	Binder Clusters
8	3	4	1.0445	0.0505	0.25	0.0705	0.6605	2.544	0.125	1.052	2.0945	1.925	0.433									308						_pos ctrls					64		ESAT-6 p51_308	1.376125	high	_pos ctrls
7	4	3	0.385	0.133	0.61	0.086	0.8465	0.522	0.074	0.365	0.517	2.504	0.051									307						_pos ctrls					64		ESAT-6 p47_307	0.85925	high	_pos ctrls
104	4	0	0.0955	0.244	0.657	0.2785	0.4055	0.135	0.057	0.0465	0.055	0.0655	-0.0015	2.828	0.3	2	13	1	384		0.5	160	100	1	2	Rv1697	Mb1723	cl_netmhciipan	3	#230	20	RTDGVVLHWIIDYWNRFSLW	36	371	Mb1723_160	0.041375	high	Binder Clusters
4	4	4	0.476	1.5535	0.251	0.1635	1.2645	0.495	0.075	1.169	2.727	0.718	0.3335									304						_pos ctrls					73		CFP10.9_304	1.236875	high	_pos ctrls
6	4	3	0.3865	0.165	0.5	0.1685	1	0.246	0.061	0.3035	0.3395	1.1805	0.04									306						_pos ctrls					64		ESAT-6 p46_306	0.465875	high	_pos ctrls
279	4	0	0.1935	0.304	1.636	0.4625	1.091	0.205	0.066	0.0455	0.0525	0.0675	-0.0025	2.449	0.3	2	10	1	76		0.55	174	99.6	1	1	Rv2001	Mb2024	cl_netmhciipan	2	#201	20	AEVHPHWIVLRTVIDVINPI	36	66	Mb3949c_174	0.04075	high	Binder Clusters
276	4	4	0.2835	0.077	0.393	0.0715	0.4815	0.566	0.072	1.1175	2.073	2.665	0.2295	6	0.15	3	20	3	203		0.55	120	100	1	2	Rv3863	Mb3893	cl_netmhciipan	3	#248	20	LAADGIINAGALIAFEKGRS	73	183	Mb3893_120	1.52125	high	Binder Clusters
126	4	4	0.323	0.4495	0.117	0.1365	1.985	0.351	0.08	1.07	2.9775	0.8845	0.3575					50	122		0.4	233		0	0	Rv2140c	Mb2164c	topsharedbinders	0	#184	20	PGGALTLVNDAGMRRYVGAA	73	102	Mb2164c_233	1.322375	high	Top Scoring Binders
10	4	4	0.7695	0.0545	1.115	0.0715	1.738	1.292	0.078	0.706	3.1705	2.0115	0.439									310						_pos ctrls					73		ESAT-6 p53_310	1.58175	high	_pos ctrls
264	4	0	0.075	0.238	0.808	0.233	0.5275	0.118	0.058	0.0555	0.141	0.069	-0.003	6.325	0.35	2	18	4	46		0.5	31	100	1	2	Rv1531	Mb1558	cl_tepitopepan	3	#261	20	MAELSIFQVLLNHPRLARTF	36	28	Mb3705_31	0.065625	high	Binder Clusters
67	4	4	0.251	0.0525	0.708	0.074	0.7915	0.686	0.083	0.8265	2.2675	1.445	0.2965	5.657	0.3	2	12	4	162		0.45	36	99.7	1	2	Rv3671c	Mb3695c	cl_tepitopepan	3	#250	20	NEAAPTWLKTVPKRLSALLN	73	150	Mb1371_36	1.208875	high	Binder Clusters
84	4	1	0.3065	0.1805	0.643	0.1505	0.985	0.342	0.069	0.1245	0.263	0.144	0.014					89	30		0.85	232		0	1	Rv1488	Mb1524	topsharedbinders	1	#117	20	LAVLVIFAIIVVAKSVALIP	45	10	Mb1524_232	0.136375	high	Top Scoring Binders
109	4	4	0.18	0.2445	0.853	0.0785	0.618	0.579	0.075	0.497	1.1675	0.9595	0.1135	3.162	0.3	2	18	1	71	pgsA2	0.45	81	100	2	2	Rv1822	Mb1853	cl_tepitopepan	4	#264	20	DWADGKIARLLNQSSRLGAL	73	53	Mb1859_81	0.684375	high	Binder Clusters
125	4	2	0.101	0.2085	0.183	0.2485	0.263	0.394	0.104	0.1215	0.5765	0.7805	0.001	9.95	0.3	3	12	11	145		0.55	103	100	2	0	Rv2129c	Mb2153c	cl_netmhciipan	2	#85	20	IDRRGYVLIVSSLAAFAAPP	55	133	Mb2153c_103	0.369875	high	Binder Clusters
9	4	4	0.2595	0.0545	1.105	0.0705	2.6335	0.929	0.117	1.7695	3.7765	3.336	0.624									309						_pos ctrls					73		ESAT-6 p52_309	2.3765	high	_pos ctrls
166	4	3	0.2785	0.052	0.901	0.0765	0.3795	0.539	0.095	0.1585	0.3705	0.6355	0.0305	3.464	0.35	2	19	3	39	rpsT	0.45	74	100	0	1	Rv2412	Mb2435	cl_tepitopepan	1	#181	20	RNKAVKSSLRTAVRAFREAA	64	20	Mb2561c_74	0.29875	high	Binder Clusters
269	5	4	0.515	0.36	0.65	0.1575	2.065	0.747	0.112	1.445	3.248	1.3425	0.383	2.828	0.3	2	19	1	197		0.5	169	100	2	0	Rv3732	Mb3759	cl_netmhciipan	2	#183	20	PYVRDGWAFVAIRLTSTDLI	82	178	Mb3759_169	1.604625	high	Binder Clusters
5	5	4	0.5755	0.293	0.531	0.123	3.698	1.146	0.087	1.403	3.532	1.9335	0.5435									305						_pos ctrls					82		ESAT-6 p45_305	1.853	high	_pos ctrls
193	5	1	0.0965	0.5815	1.223	0.7365	0.3305	0.753	0.058	0.063	0.104	0.1875	-0.011	4.899	0.3	2	15	2	311	add	0.2	131	100	3	2	Rv3313c	Mb3342c	cl_netmhciipan	5	#49	20	RVTVNTDNRLMSDTSMSLEM	55	296	Mb2983c_131	0.085875	high	Binder Clusters
167	5	2	0.203	0.3145	0.982	0.3195	0.343	0.276	0.106	0.1175	0.6785	0.9725	-0.002	2.449	0.25	2	15	1	77	tmk	0.55	179	100	0	2	Rv3247c	Mb3275c	cl_netmhciipan	2	#18	20	ASSVYAMATLFALDRAGAVH	64	62	Mb2567c_179	0.441625	high	Binder Clusters
2	5	3	0.9465	1.7805	0.39	2.1065	0.12	0.587	0.135	0.299	0.89	3.608	0.0395									302						_pos ctrls					73		CFP10.7_302	1.209125	high	_pos ctrls
59	5	2	0.153	0.589	1.126	0.4675	0.956	0.302	0.103	0.048	0.2305	0.4175	0.006	6.928	0.35	4	18	3	362	corA	0.5	110	99.5	1	1	Rv1239c	Mb1271c	cl_netmhciipan	2	#87	20	PTVIGGMVLICLFLYHVFRN	64	344	Mb1271c_110	0.1755	high	Binder Clusters
0	1		0.1565	0.578	0.2145	0.1425	0.104	0.075	0.0485						0.35				153		0.35	Mb2635c_69	100			Rv2603c	Mb2635c	random binders	1	251		LFSRKGVVTLEKNGLTEDDV	9	133			low	Random Binders (Control)
1	0		0.136	0.1565	0.0685	0.0595	0.0565	0.064	0.0485						0.25				111		0.55	Mb1266_85	100			Rv1234	Mb1266	random binders	2	175		SGAWLGLFIGLVLGFFSPNP	0	91			low	Random Binders (Control)

2	0	0.128	0.068	0.111	0.05	0.073	0.061	0.049	0.3	392	0.45	Mb1687_12	100	Rv1659	Mb1687	random binders	1	470	TDLAEWLVRQGVPFRSAHEA	0	372	low	Random Binders (Control)
3	0	0.17	0.048	0.181	0.127	0.083	0.0485	0.0485	0.2	398	0.3	Mb1482c_83	100	Rv1447c	Mb1482c	random binders	2	514	FRRAPHLPFDATMTDELGTN	0	378	low	Random Binders (Control)
4	1	0.1815	0.295	0.345	0.049	0.1525	0.0505	0.051	0.3	62	0.4	Mb0358_87	100	Rv0350	Mb0358	random binders	0	625	ARNGEVLVGQPAKNQAVTNV	9	42	low	Random Binders (Control)
5	0	0.166	0.136	0.292	0.151	0.0945	0.0485	0.0475	0.4	228	0.5	Mb0069_90	99.7	Rv0068	Mb0069	random binders	6	303	VSNTEVVRNMPRPLVAVAAI	0	208	low	Random Binders (Control)
6	0	0.2005	0.154	0.632	0.075	0.1005	0.0505	0.052	0.45	94	0.55	Mb3835c_62	99.8	Rv3805c	Mb3835c	random binders	2	627	GPMRLEYVALALAMVLSLLG	0	74	low	Random Binders (Control)
7	1	0.2085	0.2515	0.373	0.173	0.1935	0.229	0.0505	0.3	20	0.4	Mb2917_21	100	Rv2893	Mb2917	random binders	3	325	MTVASTAHHTRRLRFGLAAP	9	0	low	Random Binders (Control)
8	1	0.211	0.1745	0.369	0.0635	0.0885	0.05	0.0475	0.45	45	0.4	Mb3072c_64	100	Rv3046c	Mb3072c	random binders	2	124	VPGPDRVALLSLLRSTPLTE	9	25	low	Random Binders (Control)
9	0	0.1665	0.054	0.2005	0.047	0.098	0.0505	0.0465	0.25	49	0.4	Mb1444_51	99.4	Rv1409	Mb1444	random binders	0	339	KPPVGAVIVDPNGRIVGAGG	0	29	low	Random Binders (Control)
10	0	0.1275	0.0455	0.1315	0.0475	0.068	0.0595	0.045	0.25	122	0.4	Mb3507_66	98.1	Rv3479	Mb3507	random binders	2	426	KKTPSLLYGDERIFAALAKR	0	102	low	Random Binders (Control)
11	0	0.151	0.048	0.305	0.057	0.055	0.048	0.045	0.45	304	0.55	Mb1655_37	100	Rv1629	Mb1655	random binders	0	904	LFDDLEFRVLRDRLFDTLAA	0	284	low	Random Binders (Control)
12	0	0.152	0.047	0.0515	0.083	0.0545	0.047	0.0465	0.1	28	0.35	Mb3831c_93	99.8	Rv3801c	Mb3831c	random binders	0	637	MAYHNPFIVNGKIRFPANTN	0	8	low	Random Binders (Control)
13	1	0.1335	0.0455	0.0795	0.0635	0.301	0.046	0.0515	0.35	1167	0.4	Mb0687_48	100	Rv0668	Mb0687	random binders	1	1316	SIHDKHIEVIVRQMLRRVTI	9	1147	low	Random Binders (Control)
14	0	0.127	0.0465	0.2825	0.109	0.0745	0.05	0.0475	0.25	73	0.55	Mb1707_6	99.6	Rv1680	Mb1707	random binders	3	274	IAGHIDIAWNTNLAYVRTVL	0	53	low	Random Binders (Control)
15	0	0.1385	0.05	0.5415	0.071	0.069	0.063	0.049	0.25	431	0.5	Mb0272c_2	100	Rv0266c	Mb0272c	random binders	0	1209	QVAEGYLRIAVANMANAVKK	0	411	low	Random Binders (Control)
16	1	0.2205	0.282	0.276	0.0495	0.0515	0.0555	0.0485	0.45	195	0.4	Mb2183c_28	100	Rv2159c	Mb2183c	random binders	0	344	LMRRAGGLVFARKVRAEHRP	9	175	low	Random Binders (Control)
17	0	0.154	0.084	0.301	0.0535	0.079	0.0475	0.0475	0.25	102	0.4	Mb2935_56	100	Rv2911	Mb2935	random binders	0	291	LDLNSTVVADVADTQAECNC	0	82	low	Random Binders (Control)
18	0	0.119	0.0475	0.4035	0.072	0.0645	0.054	0.047	0.45	334	0.55	Mb3808_1	100	Rv3779	Mb3808	random binders	1	666	WWLFEALRHPVRSRLADLLT	0	314	low	Random Binders (Control)
19	1	0.1675	0.245	0.5925	0.0895	0.175	0.0535	0.0445	0.25	3793	0.5	Mb2074c_47	99.4	Rv2048c	Mb2074c	random binders	0	4151	DRAALAKVIADIPVQHPLSG	9	3773	low	Random Binders (Control)
20	0	0.125	0.046	0.0585	0.0865	0.0995	0.046	0.0455	0.4	379	0.55	Mb2224_43	100	Rv2201	Mb2224	random binders	0	652	DPALVPLFFVAREARKHVKV	0	359	low	Random Binders (Control)
21	0	0.192	0.078	0.1325	0.047	0.052	0.047	0.046	0.45	200	0.4	Mb3298_10	100	Rv3270	Mb3298	random binders	1	718	WLLNIGEYLQDLTLRRTRRA	0	180	low	Random Binders (Control)
22	1	0.117	0.0565	1.1735	0.086	0.1135	0.059	0.0485	0.35	39	0.55	Mb2443c_24	100	Rv2420c	Mb2443c	random binders	0	126	AKLADDVVVIDVSGQLVITD	9	19	low	Random Binders (Control)
23	1	0.1605	0.257	0.438	0.0645	0.07	0.058	0.0475	0.25	1313	0.3	Mb2958_80	100	Rv2933	Mb2958	random binders	1	2188	RTSWDGIVVVCPPRANDESM	9	1293	low	Random Binders (Control)
24	2	0.19	0.251	0.18	0.049	0.292	0.0505	0.048	0.3	645	0.3	Mb3175_39	99.8	Rv3151	Mb3175	random binders	0	806	VNWEGRYRTFEPALRGSTLQ	18	625	low	Random Binders (Control)
25	0	0.12	0.108	0.251	0.058	0.059	0.0505	0.046	0.5	153	0.4	Mb3811_70	100	Rv3782	Mb3811	random binders	0	304	FPLRRGLVWRRRASELRTEA	0	133	low	Random Binders (Control)
26	0	0.16	0.0465	0.0835	0.0595	0.0805	0.0475	0.045	0.3	481	0.5	Mb3324_19	99.8	Rv3296	Mb3324	random binders	8	1513	DAWFDTVRRAAPFATLPRSL	0	461	low	Random Binders (Control)
27	1	0.1235	0.065	0.8995	0.1045	0.047	0.21	0.046	0.4	331	0.35	Mb1780_32	99.8	Rv1751	Mb1780	random binders	0	460	DEVKHLNVKVNRLRRWHIDG	9	311	low	Random Binders (Control)
28	0	0.132	0.135	0.129	0.0785	0.164	0.047	0.047	0.25	61	0.35	Mb3133_88	99.6	Rv3106	Mb3133	random binders	0	456	LPTPWGLVRSGVAPDHPKIK	0	41	low	Random Binders (Control)
29	0	0.1285	0.0485	0.062	0.049	0.056	0.0465	0.048	0.3	91	0.5	Mb1990_45	100	Rv1955	Mb1990	random binders	2	125	ANNHFRVLFFRWGQHPVALT	0	71	low	Random Binders (Control)
30	1	0.1305	0.0525	0.5695	0.053	0.304	0.0495	0.0465	0.2	136	0.25	Mb1533_55	100	Rv1496	Mb1533	random binders	2	334	VHPNAYIRPSPTSGTLGGVT	9	116	low	Random Binders (Control)
31	0	0.165	0.074	0.293	0.101	0.1005	0.0675	0.047	0.3	659	0.5	Mb1563_27	99.9	Rv1536	Mb1563	random binders	2	1041	DAMRWFLMASPILRGGNLIV	0	639	low	Random Binders (Control)
32	1	0.1375	0.392	0.706	0.0495	0.0655	0.0525	0.0475	0.35	264	0.6	Mb3917c_95	99.7	Rv3887c	Mb3917c	random binders	2	288	KRWQTAVVTAVVTVCGILAA	9	244	low	Random Binders (Control)
33	0	0.18	0.1405	0.485	0.0915	0.129	0.0855	0.047	0.25	109	0.5	Mb1612c_26	100	Rv1586c	Mb1612c	random binders	2	469	PIELEAFMSLADEKRLALAT	0	89	low	Random Binders (Control)
34	0	0.1655	0.046	0.2495	0.0605	0.0635	0.0515	0.046	0.25	370	0.2	Mb2875c_78	99.7	Rv2850c	Mb2875c	random binders	0	629	RPASPRLRSSPRPSAPPSKI	0	350	low	Random Binders (Control)
35	0	0.144	0.0965	0.1135	0.0535	0.089	0.049	0.046	0.05	83	0.3	Mb1000c_58	100	Rv0975c	Mb1000c	random binders	0	349	GDGADPVIICEEMHQAGAPG	0	63	low	Random Binders (Control)
36	0	0.1555	0.049	0.194	0.0465	0.106	0.0485	0.0465	0.3	44	0.45	Mb1034c_4	99.8	Rv1007c	Mb1034c	random binders	0	519	YIATDAIARFKRLDRYDVRF	0	24	low	Random Binders (Control)
37	2	0.178	0.052	0.751	0.048	0.076	0.054	0.1965	0.3	147	0.5	Mb2145c_54	100	Rv2121c	Mb2145c	random binders	1	284	GIEATVIRLDGAVEISVQLG	18	127	low	Random Binders (Control)
38	0	0.2125	0.0485	0.2315	0.079	0.059	0.049	0.047	0.35	297	0.8	Mb0552_79	99.8	Rv0538	Mb0552	random binders	2	548	LLIGNAVVAFAVIGFASLAV	0	277	low	Random Binders (Control)
39	0	0.1905	0.05	0.4225	0.05	0.163	0.051	0.0515	0.15	534	0.3	Mb1868c_73	100	Rv1837c	Mb1868c	random binders	1	741	MWTMTELMADMVETKIAQPR	0	514	low	Random Binders (Control)
40	1	0.1055	0.2865	0.0765	0.0625	0.055	0.051	0.0475	0.3	67	0.5	Mb2172c_41	100	Rv2148c	Mb2172c	random binders	1	258	FFPATDVAILFRLGCRSVGE	9	47	low	Random Binders (Control)
41	1	0.1685	0.1935	0.218	0.0605	0.112	0.0565	0.0495	0.35	311	0.45	Mb0791_5	100	Rv0768	Mb0791	random binders	1	489	GCAITTRLVVPRARYEEAVA	9	291	low	Random Binders (Control)
42	0	0.127	0.0685	0.157	0.047	0.24	0.052	0.048	0.35	100	0.3	Mb3854c_20	100	Rv3824c	Mb3854c	random binders	3	511	LRAMTYVINAHLRRHDTYRS	0	80	low	Random Binders (Control)
43	0	0.13	0.054	0.2845	0.0625	0.0545	0.0495	0.047	0.2	339	0.5	Mb1936_7	100	Rv1901	Mb1936	random binders	0	430	NEAKAQLLGVDPALIEAHGA	0	319	low	Random Binders (Control)
44	0	0.1235	0.0475	0.4145	0.105	0.1435	0.0505	0.045	0.3	70	0.65	Mb3182_38	100	Rv3158	Mb3182	random binders	2	531	AVALIAVIVVARSIHGSGHA	0	50	low	Random Binders (Control)
45	0	0.139	0.135	0.2	0.0535	0.1005	0.139	0.05	0.5	782	0.35	Mb3324_18	99.8	Rv3296	Mb3324	random binders	2	1513	RFRESAARALLLPRRHPGRR	0	762	low	Random Binders (Control)
46	1	0.1465	0.186	0.3845	0.0455	0.0575	0.062	0.048	0.4	429	0.3	Mb2250_94	99.6	Rv2226	Mb2250	random binders	0	513	HHRDEALHLIRKRAKRLRYT	9	409	low	Random Binders (Control)
47	1	0.144	0.212	0.565	0.122	0.0615	0.054	0.0525	0.2	139	0.4	Mb1637_76	100	Rv1611	Mb1637	random binders	0	272	DFVVQPYQIHEARAHGADML	9	119	low	Random Binders (Control)
48	1	0.121	0.14	0.8825	0.0495	0.141	0.05	0.0475	0.35	1691	0.4	Mb2965c_72	100	Rv2940c	Mb2965c	random binders	2	2111	FRRGLTFYYLDLALMSVTQP	9	1671	low	Random Binders (Control)
49	0	0.1015	0.113	0.3085	0.055	0.181	0.055	0.0465	0.4	82	0.45	Mb1138c_60	100	Rv1108c	Mb1138c	random binders	0	415	TCSRDLVLSAPVKLAEGVQV	0	62	low	Random Binders (Control)
50	0	0.0745	0.087	0.322	0.0515	0.056	0.0525	0.048	0.2	363	0.7	Mb2895c_52	100	Rv2870c	Mb2895c	random binders	0	413	FPAIVGIIADVLHAADQWAV	0	343	low	Random Binders (Control)
51	1	0.131	0.315	0.073	0.1305	0.183	0.053	0.049	0.25	220	0.35	Mb0338_53	100	Rv0331	Mb0338	random binders	1	388	PEVGEALVSMLKDHGVGFHP	9	200	low	Random Binders (Control)
52	0	0.1115	0.152	0.0695	0.0475	0.066	0.053	0.054	0.2	204	0.4	Mb3374_84	100	Rv3342	Mb3374	random binders	3	243	PQALIDLVASRSYCITSPAQ	0	184	low	Random Binders (Control)
53	0	0.164	0.136	0.1805	0.048	0.071	0.0525	0.046	0.35	403	0.55	Mb3925c_82	99.8	Rv3895c	Mb3925c	random binders	2	495	DRAPASVVATQVLVLPGAAN	0	383	low	Random Binders (Control)
54	1	0.1255	0.633	0.3785	0.05	0.08	0.2055	0.0465	0.3	197	0.4	Mb0705_13	99.6	Rv0686	Mb0705	random binders	1	265	GPLMEILQILKANNVPLNRM	9	177	low	Random Binders (Control)
55	0	0.14	0.165	0.3325	0.0845	0.0755	0.0575	0.0485	0.45	218	0.7	Mb0162_59	100	Rv0157	Mb0162	random binders	1	475	WWMIGLLVAAGVLGLMVVLP	0	198	low	Random Binders (Control)
56	0	0.0985	0.1135	0.0845	0.073	0.075	0.05	0.0465	0.35	603	0.25	Mb1591c_31	100	Rv1564c	Mb1591c	random binders	0	721	TTLRKNHKVFRRRRFFEGEP	0	583	low	Random Binders (Control)
57	1	0.1055	0.2365	0.562	0.164	0.0525	0.048	0.0495	0.2	251	0.4	Mb2286_74	100	Rv2263	Mb2286	random binders	3	317	RLYKTSWRFAPFLWQEIEEG	9	231	low	Random Binders (Control)
58	1	0.177	0.175	0.1985	0.1555	0.05	0.085	0.0475	0	809	0.4	Mb1511c_3	100	Rv1475c	Mb1511c	random binders	4	943	PGGPQAFIYDAAQNYAAQHI	9	789	low	Random Binders (Control)
59	1	0.1255	0.1885	0.053	0.0465	0.0535	0.1315	0.0475	0.1	321	0.4	Mb3180_11	99.8	Rv3156	Mb3180	random binders	3	633	GCAKDDIKRALAASTISQIG	9	301	low	Random Binders (Control)
60	1	0.203	0.2875	0.151	0.072	0.0735	0.052	0.0485	0.4	293	0.6	Mb1615_86	100	Rv1589	Mb1615	random binders	5	349	LKAVAAFRLALPRTMLRFAG	9	273	low	Random Binders (Control)
61	0	0.155	0.1695	0.3125	0.076	0.062	0.2745	0.0475	0.3	21	0.25	Mb2967_22	100	Rv2942	Mb2967	random binders	1	920	PSPAGRLHRIRYIRLKKSSP	0	1	low	Random Binders (Control)
62	0	0.139	0.101	0.2425	0.049	0.0575	0.0485	0.0475	0.3	72	0.55	Mb2052c_14	99.8	Rv2027c	Mb2052c	random binders	1	573	ILAITSGLKLDATLRAIVHT	0	52	low	Random Binders (Control)
63	0	0.108	0.0895	0.1155	0.045	0.0535	0.0515	0.046	0.25	38	0.4	Mb3301_15	100	Rv3273	Mb3301	random binders	3	764	APASRSQWMLANLRHDVPAS	0	18	low	Random Binders (Control)
64	0	0.1245	0.054	0.245	0.047	0.185	0.052	0.044	0.25	33	0.25	Mb1046c_81	99.6	Rv1018c	Mb1046c	random binders	3	495	AGPGTRMRSDTPKVLHTLAG	0	13	low	Random Binders (Control)
65	0	0.1405	0.0495	0.0475	0.0445	0.0595	0.0465	0.046	0.4	81	0.55	Mb0243_68	99.7	Rv0237	Mb0243	random binders	0	388	RDKLAQLLVVGVRDAADAQA	0	61	low	Random Binders (Control)
66	0	0.1065	0.0685	0.257	0.0545	0.0555	0.1275	0.048	0.3	731	0.45	Mb0433c_92	99.9	Rv0425c	Mb0433c	random binders	0	1539	GSPVDAVMVGSVLTGNSILA	0	711	low	Random Binders (Control)
67	0	0.1445	0.0675	0.4695	0.057	0.077	0.0515	0.0495	0.45	206	0.35	Mb1062c_25	100	Rv1033c	Mb1062c	random binders	2	257	TEFELLRFLMRNPRRALSRT	0	186	low	Random Binders (Control)
68	1	0.188	0.438	0.1145	0.0465	0.114	0.053	0.0505	0.3	28	0.4	Mb2172c_40	100	Rv2148c	Mb2172c	random binders	2	258	PDRESELTHALAAMRSRLAA	9	8	low	Random Binders (Control)
69	1	0.119	0.255	0.108	0.049	0.0665	0.049	0.047	0.4	214	0.45	Mb0868_57	99.8	Rv0845	Mb0868	random binders	3	425	ATALMVVRIEERHTRSVAGL	9	194	low	Random Binders (Control)
70	0	0.1295	0.045	0.0935	0.057	0.054	0.0495	0.0465	0.45	110	0.45	Mb3835c_61	99.8	Rv3805c	Mb3835c	random binders	1	627	SLLGMVLLMLGTGRLYAPSL	0	90	low	Random Binders (Control)
71	0	0.197	0.046	0.1625	0.064	0.068	0.0485	0.0465	0.35	1691	0.45	Mb3855c_49	100	Rv3825c	Mb3855c	random binders	5	2126	TKLGLFPFRRNLSFYAVDLG	0	1671	low	Random Binders (Control)
72	0	0.1325	0.0445	0.339	0.1295	0.061	0.0495	0.045	0.25	917	0.4	Mb0042_65	100	Rv0041	Mb0042	random binders	0	969	PKADAAYLVDETVEYPVQVN	0	897	low	Random Binders (Control)
73	1	0.1295	0.1985	0.2065	0.073	0.053	0.063	0.045	0.4	94	0.5	Mb2195_89	100	Rv2173	Mb2195	random binders	0	352	LLHAWALVHDDLIDRSATRR	9	74	low	Random Binders (Control)
74	1	0.1405	0.445	0.2585	0.099	0.085	0.048	0.0445	0.35	135	0.55	Mb1199c_30	100	Rv1167c	Mb1199c	random binders	1	201	LLHEPELRAIFINSVWAPRL	9	115	low	Random Binders (Control)
75	1	0.1525	0.182	0.1375	0.046	0.05	0.049	0.046	0.15	210	0.3	Mb2321c_36	100	Rv2299c	Mb2321c	random binders	1	647	HDYTSEWKIRNLVKKYSDFI	9	190	low	Random Binders (Control)
76	1	0.136	0.221	0.2295	0.06	0.106	0.06	0.0505	0.2	64	0.4	Mb1869c_50	100	Rv1838c	Mb1869c	random binders	0	131	QEICHRYVAIKRREAIQPAF	9	44	low	Random Binders (Control)
77	1	0.142	0.1985	0.105	0.048	0.0615	0.0565	0.0485	0.25	227	0.45	Mb0900c_77	99.8	Rv0876c	Mb0900c	random binders	4	548	YPCALAMMVFSKSFSVLRSA	9	207	low	Random Binders (Control)
78	0	0.0465	0.046	0.0475	0.047	0.0475	0.055	0.048	0.4	1040	0.25	Mb2960_23	99.9	Rv2935	Mb2960	random binders	1	1488	VPPNVTYFLDRGLRDTGRCR	0	1020	low	Random Binders (Control)
79	0	0.1935	0.045	0.254	0.078	0.0865	0.0475	0.047	0.2	93	0.4	Mb0250c_46	99.7	Rv0244c	Mb0250c	random binders	1	611	VMLPESFKKSVNAMLEAGWD	0	73	low	Random Binders (Control)
80	0	0.1165	0.167	0.374	0.047	0.065	0.048	0.0465	0.35	194	0.65	Mb1625_91	100	Rv1599	Mb1625	random binders	1	438	LAAARLLGVDEVWAVGGAQA	0	174	low	Random Binders (Control)
81	0	0.1245	0.047	0.059	0.0865	0.0515	0.0465	0.045	0.65	322	0.45	Mb2585c_35	99.9	Rv2555c	Mb2585c	random binders	1	904	GRGYVLRRLLRRVIRSAKLL	0	302	low	Random Binders (Control)
82	0	0.1635	0.0495	0.505	0.047	0.1075	0.048	0.047	0.35	26	0.3	Mb3265c_9	100	Rv3237c	Mb3265c	random binders	0	160	LLPGVGLRYEFTSYRGDRIG	0	6	low	Random Binders (Control)
83	0	0.1385	0.1	0.073	0.078	0.0615	0.05	0.0475	0.3	175	0.45	Mb0061_8	100	Rv0060	Mb0061	random binders	2	352	IEGVEGLRMTWGRAVILEAM	0	155	low	Random Binders (Control)
84	0	0.276	0.0725	0.3365	0.068	0.1045	0.085	0.05	0.25	411	0.6	Mb2709c_63	100	Rv2690c	Mb2709c	random binders	2	657	AELTALIQLYIVGVFISFTM	0	391	low	Random Binders (Control)
85	0	0.1175	0.046	0.187	0.0485	0.0645	0.0555	0.0485	0	313	0.4	Mb2618c_71	99.8	Rv2587c	Mb2618c	random binders	2	573	KGPAANIWADYTAAHIGTQT	0	293	low	Random Binders (Control)
86	0	0.1225	0.161	0.08	0.0485	0.069	0.0625	0.051	0.5	136	0.85	Mb1867c_67	99.7	Rv1836c	Mb1867c	random binders	3	677	IGVIVALVAVVVMVAGVILW	0	116	low	Random Binders (Control)
87	0	0.2635	0.0455	0.1525	0.0495	0.1345	0.0525	0.049	0.35	385	0.45	Mb2306_34	100	Rv2285	Mb2306	random binders	1	445	FTVSNVVLSKDPLYLSGAKL	0	365	low	Random Binders (Control)
88	0	0.137	0.0645	0.127	0.0455	0.0595	0.049	0.0475	0.45	97	0.6	Mb0825c_75	100	Rv0802c	Mb0825c	random binders	0	218	WSLPLAVLVDGRAVGVQALS	0	77	low	Random Binders (Control)
89	1	0.1505	0.0515	1.097	0.058	0.09	0.059	0.0485	0.3	340	0.45	Mb3035c_17	100	Rv3010c	Mb3035c	random binders	0	343	ADAVRKLKLVPQSRYDDAAA	9	320	low	Random Binders (Control)
90	0	0.122	0.0475	0.3025	0.0705	0.0515	0.05	0.0485	0.4	377	0.35	Mb2877c_42	100	Rv2852c	Mb2877c	random binders	3	493	NYLISQLRLSEPERVSALRE	0	357	low	Random Binders (Control)
91	0	0.1485	0.05	0.234	0.051	0.065	0.0525	0.0475	0.35	467	0.4	Mb2073c_33	100	Rv2047c	Mb2073c	random binders	1	854	SPRDTDAYVADVDRLERLAF	0	447	low	Random Binders (Control)
92	0	0.149	0.0515	0.0695	0.1425	0.07	0.0505	0.0505	0.35	107	0.4	Mb0182_29	99.4	Rv0176	Mb0182	random binders	2	322	GRALTGIRVVRRDGSAIGPW	0	87	low	Random Binders (Control)
93	0	0.101	0.0525	0.098	0.052	0.0925	0.0765	0.0485	0.45	53	0.7	Mb3301_16	100	Rv3273	Mb3301	random binders	3	764	DVPASLVVFLVALPLSLGIA	0	33	low	Random Binders (Control)
